# Plasticity-Induced Heating: Revisiting the Energy-Based Variational Model

**DOI:** 10.3390/ma17051078

**Published:** 2024-02-26

**Authors:** Christoph Hartmann, Michael Obermeyer

**Affiliations:** Chair of Metal Forming and Casting, Technical University of Munich, Walther-Meissner-Strasse 4, 85748 Garching near Munich, Germany

**Keywords:** energy-based variational model, temperature evolution, plasticity-induced heating, Taylor–Quinney coefficient, thermo-visco-plastic model

## Abstract

Temperature evolution during plastic deformation is of great importance for the design of manufacturing processes, as well as for the analysis and prediction of tool wear. However, the results from experimental- and numerical-type research are still often contradictory. In this paper, we analyze methods for estimating plasticity-induced heating directly from displacement fields that can be recorded during experiments or extracted from simulation results. In terms of computational methodology, the thermodynamically motivated energy-based variational formulation of the coupled thermo-mechanical boundary-value problem is adapted to the problem at hand. Since an analysis of this variational formulation exhibits challenges and distinct inconsistencies with respect to the problem at hand, an alternative approach is proposed. This alternative approach is essentially a purely thermal finite element simulation, and it is conducted using a heat source term that is empirically based on the fraction of irreversible deformation work converted to heat. Our approach estimates plasticity-induced heating based on the strain and strain rate data derived from displacement fields. We therefore incorporate thermo-visco-plastic constitutive behavior (Johnson–Cook) with a thermodynamically motivated model that specifies the fraction of plastic work converted to heat (the Taylor–Quinney coefficient).

## 1. Introduction

When a metallic material is subjected to an irreversible deformation, only a part of the deformation work is dissipated as heat [1,2]. The rest is stored as potential energy in the crystal lattice structure of the material. Since the storage of potential energy depends on various state variables, of which temperature is an obvious and significant influence, it is common to distinguish between cold and hot work. In the case of hot work, the temperature has to be above a certain limit, which is specific to each material, so that very little or no potential energy is stored. For the process of cold work, on the other hand, this the storage of energy is characteristic [3]. Thus, it is quite common to refer to the stored potential energy as the stored energy of cold work [4]. However, in the derivation of the variational model in Section 4, no explicit distinction is made between hot and cold work. Instead, a single temperature-dependent term for the stored potential energy is introduced. As a side note, thermoelastic phenomena also affect the temperature evolution during deformation. Due to the Joule–Thomson effect, elastic deformation can result in either a heating or cooling of the workpiece [5]. In the present work, the aforementioned phenomenon or degradation is not considered. Experimental studies have described the influence of elastic deformation on the temperature evolution as negligible, and this assumption is supported in the results of [3,4,6].

On a microscopic scale, the storage of potential energy is a result of the creation and rearrangement of crystal imperfections, namely dislocations, point defects, stacking faults and twins [4]. These crystal imperfections cause a local distortion of the lattice resulting in a microstressed state and thus store energy within the lattice structure of the material [3]. The dissipation during the process of irreversible deformation is mainly caused by the generation, movement and annihilation of point defects and dislocations [7,8]. Which of them, the point defects or the dislocations, dominates the heat generation depends on the material, especially on the structure of the crystal lattice, the temperature and the deformation state [3]. While the microscopic processes are revealing and allow a better understanding of the overall process, they will not be elaborated on as this work advocates a macroscopic approach.

## 2. State-of-the-Art Works

As introduced in Section 1, only a fraction of the work on irreversible deformation, which is more commonly referred to as plastic work, is converted into heat. What fraction of the plastic work has actually dissipated is a crucial assumption for calculating the temperature evolution, for example. Commonly referred to as β and called the Taylor–Quinney coefficient, the calculations required to determine the fraction of plastic work that is converted to heat can be traced back to Taylor and Quinney [2]. In their pioneering work, they found that β is typically between 0.8 and 1.0.

It has been common to assume that the coefficient β is a constant, e.g., as in [9,10,11,12,13]. However, most of this information was obtained from quasi-static experiments, which also showed significant discrepancies in the data for nominally identical materials [4]. Experimental investigations, e.g., as in [6,14,15,16,17], also clearly question the limited accuracy of the assumption. Thus, the assumption that the Taylor–Quinney coefficient (TQC) is a constant is an “approximation of dubious validity” [4].

Leaving this assumption aside, there are two general types of approaches to modeling β. These are either approaches developed from a macroscopic point of view, e.g., as in [4,18,19,20] or a microscopic point of view based on the above-mentioned generation and evolution of crystal defects, e.g., as in [21,22,23]. However, even the more prominent ones, such as the model derived by Rosakis et al. [4], have some significant flaws. The model created by Rosakis et al. [4] is discussed in more detail in Section 4.5.

The energy-based variational model introduced by [24], which will be the guiding framework throughout this work, refrains from explicitly modeling the TQC. Within this framework, the dissipation fraction follows directly from the definition of the state potential and a dissipation pseudo-potential [24]. This model is discussed in detail in Section 4. Strictly following mathematical conventions, the term ’coefficient’ refers only to fixed scalar parameters, i.e., the cases where β is assumed to be constant. For simplicity, all approaches to modeling the fraction of plastic work converted to heat as a function will also be referred to as TQC throughout this paper.

For the sake of brevity, we should also distinguish between two factors, $\beta_{\mathrm{int}}$ and $\beta_{\mathrm{diff}}$, as introduced by Rittel [25]. The first factor $\beta_{\mathrm{int}}$ is defined as follows

(1) $$\beta_{\mathrm{int}}=\frac{\rho c_{p}\Delta T}{A^{p}}$$

where ρ is the mass density, $c_{p}$ is the specific heat capacity, *T* is the temperature, and $A^{p}$ is the irreversible work of deformation per unit of undeformed volume. Note that the irreversible work of deformation is more commonly referred to as $W^{p}$, which has already been used to describe the stored plastic energy in this work. The factor $\beta_{\mathrm{int}}$ is exactly the factor β introduced by Taylor and Quinney [2]. The second factor $\beta_{\mathrm{diff}}$ is defined as

(2) $$\beta_{\mathrm{diff}}=\frac{\rho c_{p}\dot{T}}{{\dot{A}}^{p}},$$

and it is used for inclusion in the coupled heat equation, which is discussed in more detail in Section 4.5. In the illustrative plot of thermal work as a function of mechanical work, $\beta_{\mathrm{int}}$ is the secant slope and $\beta_{\mathrm{diff}}$ is the tangent slope [25].

Both versions have been widely used in the literature, e.g., $\beta_{\mathrm{diff}}$ by Mason et al. [16] and $\beta_{\mathrm{int}}$ by Kapoor and Nemat-Nasser [26], to name two prominent examples. However, the specific implications are rarely explicitly explained or emphasized.

In the remainder of this paper, the distinction between $\beta_{\mathrm{int}}$ and $\beta_{\mathrm{diff}}$ will be maintained. What exactly may be called the Taylor–Quinney coefficient will be clarified in a more detailed examination in Section 4.5. Furthermore, a convention will be followed that no precise differentiation is required when the term Taylor–Quinney coefficient is used without further specification. In this context, the term TQC simply refers to a factor that is used to calculate the temperature evolution by multiplying it by the corresponding work or power.

## 3. Objective

There is no general agreement on what proportion of the irreversible deformation work is dissipated in heat. This fraction, commonly referred to as the Taylor–Quinney coefficient, has been the subject of research since its introduction by Taylor and Quinney [2].

This lack of agreement is what makes the energy-based variational formulation of the coupled thermo-mechanical boundary value problem proposed by Yang et al. [24] so attractive. They claim that this variational formulation is able to model the constitutive behavior in a thermodynamically consistent way without an explicit assumption for the modeling of the Taylor-Quinney coefficient [27]. Thus, this model is the starting point for all further considerations within this work (see Section 4 for more detail).

The aim of this work is to investigate the energy-based variational model in the context of in situ measured displacement field data, which are readily available for a wide range of experiments and processes [28,29]. This becomes even more important with the increasing accuracy and precision of full-field optical measurement techniques [30,31,32,33]. The first goal of this work was to adapt the energy-based variational model to the special case of given displacement fields. This was followed by a corresponding verification of the validity of this model. Based on these results (see Section 5 for more detail), an alternative approach is presented in Section 6. Section 7 contains a summary and links the main findings of the previous sections. An outlook in Section 8 concludes the work.

## 4. The Energy-Based Variational Model

In general, calculus is a branch of mathematical analysis that deals with the real functions of functions, called functionals. Functionals are often expressed as integrals of functions and their derivatives. The goal is to find stationary functions that, apart from singularities, lead to the maxima, minima, or saddle points of the functional. Some classical problems in mathematics and physics can be solved elegantly with the help of functionals.

In this way, Yang et al. [24] derived a thermodynamically consistent variational formulation of the coupled thermo-mechanical boundary value problem for general dissipative solids, which is simply referred to in this work as the energy-based variational model (EBVM). In this section, the mathematical basis for the EBVM is established, starting from Yang et al. [24] and then accompanied by additional insights from Stainier and Ortiz [27], Stainier [34], and Su and Stainier [35]. Further, our own explanatory intermediate steps, assumptions, and modifications are also included. Separating them into a separate chapter would lead to unreasonable complication and would severely impair the overall comprehensibility. This section is divided into five subsections, of which Section 4.1 illustrates the approach for general dissipative solids and Section 4.2 treats the adaptation to the case of finite thermo-visco-plasticity. Subsequently, the Johnson–Cook plasticity model is incorporated into the EBVM framework (see Section 4.3 for more detail). In Section 4.4 the EBVM is further adapted to handle the given displacement fields. The introduction and adaptation is concluded with a more detailed examination of the TQC in Section 4.5.

### 4.1. Derivation for General Dissipative Solids

Fundamental to this framework is the assumption of a Helmholtz free energy

(3) W=U−TN

where *W* is the Helmholtz free energy per unit of undeformed volume, *U* is the internal energy per unit of undeformed volume, *T* is the absolute temperature, and *N* is the entropy per unit of undeformed volume [24,36] (pp. 112–114).

All motions and thermodynamic processes were performed by a continuum body with the reference configuration $\Omega \subset R^{dim},dim\in \left\{2,3\right\}$. The motions were described by a time-dependent deformation mapping $\varphi :\Omega \times \left[a,b\right]\mapsto R^{dim}$, where [a,b] is the corresponding time interval. The deformation gradient is introduced as F=∇φ. Furthermore, a collection Z of general internal variables was assumed, the specific form of which depended on the material class and could not be defined universally for all solids. This led to the reformulation of Equation (3) (as in [24]):

(4) W(F,T,Z)=U(F,N,Z)−TN.

Applying the Legendre–Fenchel transformation to the internal energy *U* leads to [34]

(5) $$U\left(F,N,Z\right)=\underset{T}{\sup}\;\left[NT+W\left(F,T,Z\right)\right],$$

in terms of which the equilibrium relations can be stated as

(6) $$\begin{array}{cccc} & N & & =-\frac{\partial W}{\partial T}\left(F,T,Z\right), \end{array}$$

(7) $$\begin{array}{cccc} & P^{e} & & =\frac{\partial W}{\partial F}\left(F,T,Z\right)\;\mathrm{and} \end{array}$$

(8) $$\begin{array}{cccc} & Y & & =-\frac{\partial W}{\partial Z}\left(F,T,Z\right). \end{array}$$

Therein, $P^{e}$ are the equilibrium stresses and Y is the thermodynamic driving forces that conjugate with the internal variables Z [24]. Furthermore, the first Piola–Kirchhoff stress tensor P can be split as

(9) $$P=P^{e}+P^{v}$$

where $P^{v}$ is the viscous stress. To ensure thermodynamic consistency, the first principle of thermodynamics in its local form

(10) $$\dot{U}=P\cdot \dot{F}+RQ-\mathrm{Div}H$$

must hold. Therein, *R* is the mass density per unit of undeformed volume, *Q* the distributed heat source per unit mass, and H the outward heat flux. With the theorem of Coleman and Noll [37], $T=\partial_{N}U$, and Equations (7)–(9), this can be restated as [24]

(11) $$T\dot{N}=P^{v}\cdot \dot{F}+Y\cdot \dot{Z}-\mathrm{Div}H+RQ.$$

Thus, it becomes clear that the equilibrium relations must be supplemented with an appropriate kinetic relation that allows the determination of $P^{v}$, $\dot{Z}$, and H in order to obtain a closed set of governing equations that define a well-posed initial boundary-value problem [24]. A general kinetic potential is a function $\Delta (\dot{F},\dot{Z},G,F,N,Z)$ for that [24,27]

(12) $$\begin{array}{cc} P^{v} & =\frac{\partial \Delta }{\partial \dot{F}}, \end{array}$$

(13) $$\begin{array}{cc} Y & =\frac{\partial \Delta }{\partial \dot{Z}}\;\mathrm{and} \end{array}$$

(14) $$\begin{array}{cc} -H & =\frac{\partial \Delta }{\partial G}. \end{array}$$

For example, a material with uncoupled viscosity, rate sensitivity and heat conduction has a general kinetic potential of the form

(15) Δ=ϕ*+τ*−χ,

where ϕ*, τ* and χ are the viscous potential, the kinetic potential and a Fourier potential, respectively [24]. The superscript (·)* refers to the use of the dual potential, the implications of which will be explained in Section 4.2. Regarding the heat conduction, the Fourier potential χ(G), commonly known as Biot’s dissipation function, was assumed such that [38]

(16) $$H=\partial_{G}\chi \left(G\right)$$

with

(17) $$G=-\frac{\nabla T}{T}.$$

With the above equations, the constitutive equations of continuum mechanics can be restated in a thermodynamically consistent, variational framework, as was explained Ortiz and Stainier [39] or Yang et al. [24]. This eventually leads to a power density function

(18) $$D\left(\dot{\varphi },T,\dot{N},\dot{Z}\right)=\dot{U}-\dot{N}T+\Delta \left(\frac{T}{\vartheta }\dot{F},\frac{T}{\vartheta }\dot{Z},G\left(T\right)\right)$$

where $\vartheta \left(F,N,Z\right)=\partial_{N}U$ is the equilibrium temperature in contrast to the external temperature field *T*. This distinction follows from the variational formulation. The optimality condition with respect to $\dot{N}$ yields $T=\partial_{N}U=\vartheta$ [24,27]. It is important to note that this power density function does not include inertial effects or distributed heat sources. Considering the problem at hand, namely the estimation of the temperature field evolution from displacement field data, the neglect of inertial effects seems acceptable at first. As for heat sources, of which there were none anyway, the heating of the material was due to the dissipation of plastic work. With respect to Equation (4), resulting in $\dot{U}=\dot{W}+\dot{N}T+N\dot{T}$, Equation (18) is restated as

(19) $$D\left(\dot{\varphi },T,\dot{N},\dot{Z}\right)=\dot{W}+N\dot{T}+\Delta \left(\frac{T}{\vartheta }\dot{F},\frac{T}{\vartheta }\dot{Z},G\left(T\right)\right).$$

When the above is expressed in a time-incremental framework and with integration over the whole domain, it yields the functional [24,35]

(20) $$\Phi_{n}=\int_{\Omega }\left[W_{n+1}-W_{n}+N_{n}\left(T_{n+1}-T_{n}\right)+\int_{t_{n}}^{t_{n+1}}\Delta \prime dt\prime \right]d\Omega$$

with

(21) $$\Delta \prime =\Delta \left(\frac{T_{n+1}}{T_{n}}\frac{\Delta F}{\Delta t},\frac{T_{n+1}}{T_{n}}\frac{\Delta Z}{\Delta t},-\nabla \ln\frac{T_{n+1}}{T_{n}},F\left(t\prime \right),N\left(t\prime \right),Z\left(t\prime \right)\right).$$

Again, it is important to note that this equation does not include the boundary integrals. Therefore, it only allows an implicit inclusion of the Dirichlet boundaries or an adiabatic Neumann boundary. Although the extension to general Neumann or mixed boundaries is rather straightforward [35], there will be no need for their incorporation in this work due to the examination of the adiabatic case in Section 5.

With this functional, the incremental coupled thermo-mechanical boundary value problem can be stated as a variational principle [24,35]

(22) $$\left[\varphi_{n+1},T_{n+1},Z_{n+1}\right]=\arg\;\underset{\varphi_{n+1},Z_{n+1}}{\inf}\underset{T_{n+1}}{\sup}\;\Phi_{n}\left(\varphi_{n+1},T_{n+1},Z_{n+1}\right).$$

It should be noted that, unlike [24], the variation used in this paper does not involve minimization with respect to $N_{n+1}$, which is a consequence of reformulating the Equation (18) in the manner of Equation (19). In addition, $\Phi_{n}$ does not depend on the gradient of $Z_{n+1}$, which allows for a local enforcement of the minimization [24]. Restating the functional $\Phi_{n}$ as

(23) $$\Phi_{n}=\int_{\Omega }\underset{Z_{n+1}}{\inf}\left[W_{n+1}-W_{n}+N_{n}\left(T_{n+1}-T_{n}\right)+\int_{t_{n}}^{t_{n+1}}\Delta \prime dt\prime \right]d\Omega$$

eventually yields

(24) $$\left[\varphi_{n+1},T_{n+1}\right]=\arg\;\underset{\varphi_{n+1}}{\inf}\;\underset{T_{n+1}}{\sup}\;\Phi_{n}\left(\varphi_{n+1},T_{n+1}\right).$$

This variational formulation has two major advantages over the conventional formulations of thermo-mechanical problems, and both are due to its variational nature. First, is the symmetry associated with all variational formulations; second, is the applicability of tools from optimization theory and mathematical programming [35]. Furthermore, this approach is characterized by its extensibility. The incorporation of material behavior such as thermal softening or strain hardening—as well as the physical implications of the process or experiment such as friction, damage, and fracture—can be accomplished by adding or changing the respective potentials.

### 4.2. Adjustment to Thermo-Visco-Plasticity

For metals, it is generally accepted that Helmholtz free energy is composed of elastic, plastic, and thermal energies, as expressed in [35]:

(25) $$W=W^{e}+W^{p}+W^{th}.$$

When considering the large plastic deformation, it is quite common to neglect the elastic deformation in the numerical simulations, e.g., as in Li and Peng [40] or Zhao et al. [41]. As already mentioned and justified in Section 1, this assumption will also be made. This implies not only that $W^{e}=0$, but also that $F=F^{p}$, which is the plastic deformation. The stored heat energy $W^{th}$ is defined as

(26) $$W^{th}=\rho_{0}c_{p}T\left(1-\ln\frac{T}{T_{r}}\right)$$

where $c_{p}$ is the specific heat capacity, $T_{r}$ is the reference temperature, and $\rho_{0}$ is the mass density. Note that, in Section 4.1, *R* was used for the mass density per unit of undeformed volume. This replacement emphasizes that from this point on, the common assumption of a constant mass density is made. Equation (26) corresponds to the expression used by Su [42]. It is similar to the one derived by Miehe [43] but reduced by a constant summand, which becomes obsolete anyway when using the incremental framework.

The term for the plastic energy $W^{p}$ is specific to the material model used, and it is defined in Section 4.3. Therefore, the expression for $N=\rho_{0}\eta =-\partial_{T}W$, with η as the specific entropy, must also be derived specifically for each material model.

The set of internal variables for thermo-visco-plastic materials is defined by Stainier and Ortiz [27] as $Z=\{F^{p},{\bar{\varepsilon }}^{p}\}$, where ${\bar{\varepsilon }}^{p}$ is a scalar internal variable measuring the cumulative or equivalent plastic strain. Similar to the work of Stainier [34], the plastic part of the deformation gradient $F^{p}$ in this work is replaced by another strain measure as an internal variable. However, in contrast to Stainier [34], the true plastic strain was chosen instead of the engineering strain. To the best of the authors’ knowledge, this did not affect the validity of the above variational principles as long as the Piola–Kirchhoff stress tensor P was also replaced by the Cauchy stress tensor σ, which is commonly referred to as the true stress.

The equivalent true plastic strain ${\bar{\varepsilon }}^{p}$ and the plastic part of the deformation gradient $F^{p}$ were then connected by $\varepsilon =\frac{1}{2}\ln\left(F^{T}\cdot F\right)$ and thus with neglecting the elastic strains $\varepsilon^{p}=\frac{1}{2}\ln\left(F^{pT}\cdot F^{p}\right)$ and the rate relation via the classical von Mises ($J_{2}$) plasticity, as shown in [35],

(27) $${\dot{\bar{\varepsilon }}}^{p}=\sqrt{\frac{2}{3}{\dot{\varepsilon }}^{p}\cdot {\dot{\varepsilon }}^{p}}.$$

However, under the assumption that the elastic strains are negligible, $\varepsilon^{p}$ is not needed as an internal variable to calculate the flow direction and thus the decomposition of F into $F^{p}$ and $F^{e}$ and ε into $\varepsilon^{p}$ and $\varepsilon^{e}$, respectively. As such, the reduction to ${\bar{\varepsilon }}^{p}$ as the only internal variable was acceptable.

Still using the assumption of uncoupled potentials as in Equation (15), the general kinetic potential of thermo-visco-plastic materials is defined as Δ=ψ*+χ, where ψ* is a dual dissipation pseudo-potential [27]. This is in agreement with [44], who state that the constitutive behavior of irreversible processes is governed by two potentials: a state potential and a dissipation pseudo-potential. Although it is common to denote the heat conduction potential as part of the dissipation pseudo-potential [45] (pp. 61–65), χ will be treated separately for the sake of clarity when considering $\partial_{\dot{Z}}\chi \left(G\right)=\partial_{{\dot{\bar{\varepsilon }}}^{p}}\chi \left(G\right)=0$. Furthermore, this work is restricted to isotropic materials with thermal conductivity λ, thus leading to an exemplary Fourier potential in the style of [35].

(28) $$\chi \left(G_{n+1},T_{n}\right)=\frac{1}{2}\lambda T_{n}G_{n+1}\cdot G_{n+1}.$$

For the dual dissipation pseudopotential ψ*, the starting point is the traditional approach of deriving the evolution of the internal variable ${\bar{\varepsilon }}^{p}$ from a dissipation pseudopotential ψ(Y) as ${\dot{\bar{\varepsilon }}}^{p}=\partial_{Y}\psi$. Note that *Y* is still the force thermodynamically conjugate to the internal variable, but with the assumption $Z={\bar{\varepsilon }}^{p}$, now a scalar. By means of a Legendre–Fenchel transformation, the dual dissipation pseudo-potential can be defined as $\psi *\left({\dot{\bar{\varepsilon }}}^{p}\right)=\sup_{Y}\left[Y{\dot{\bar{\varepsilon }}}^{p}-\psi \left(Y\right)\right]$ or—alternatively and assuming that ${\dot{\bar{\varepsilon }}}^{p}$ is determined only by the local thermodynamic state, as

(29) $$Y=\frac{\partial \psi *}{\partial {\dot{\bar{\varepsilon }}}^{p}}\left({\dot{\bar{\varepsilon }}}^{p},{\bar{\varepsilon }}^{p},T\right).$$

Note that this is consistent with Equation (13). Also note that ψ must be defined convexly in *Y*. The convexity of ψ(Y) then implies the convexity of $\psi *\left({\dot{\bar{\varepsilon }}}^{p}\right)$, which ensures the positivity of the intrinsic dissipation [27]. This positivity is needed to ensure the verification of the Clausius–Duhem inequality, which is commonly referred to as the second principle of thermodynamics [24].

The specific form of ψ* again depends on the material model used and is therefore introduced in Section 4.3.

Regardless of its specific form, there are several ways to integrate ψ* or χ as part of $\int_{t_{n}}^{t_{n+1}}\Delta \prime dt\prime$ in Equation (23). A fairly straightforward approach to integrating χ would be to use Su and Stainier [35] as

(30) $$\int_{t_{n}}^{t_{n+1}}\chi \prime dt\prime \approx \Delta t\;\chi \left(G_{n+1},T_{n})\right)$$

which is similar but not identical to the use of an implicitly computed right Riemann sum. For the integration of ψ*, however, a more sophisticated and accurate approach was used, as was suggested by Stainier [46],

(31) $$\begin{array}{cc} \int_{t_{n}}^{t_{n+1}}\psi *\prime dt\prime \; & =\int_{t_{n}}^{t_{n+1}}\psi *\left(\frac{T_{n+1}}{T_{n}}\frac{\Delta {\bar{\varepsilon }}^{p}}{\Delta t},{\bar{\varepsilon }}^{p}\left(t\prime \right),T\left(t\prime \right)\right)dt\prime \\ \; & \approx \Delta t[\frac{T_{n}}{T_{n+1}}\psi *\left(\frac{T_{n+1}}{T_{n}}\frac{\Delta {\bar{\varepsilon }}^{p}}{\Delta t},{\bar{\varepsilon }}_{n+\alpha }^{p},T_{n}\right)\\ \; & +\frac{T_{n+1}-T_{n}}{T_{n+1}}\psi *\left(\frac{T_{n+1}}{T_{n}}\frac{\Delta {\bar{\varepsilon }}^{p}}{\Delta t},{\bar{\varepsilon }}_{n+\alpha }^{p},T_{n+\alpha }\right)]. \end{array}$$

Here the strain and temperature are calculated as

(32) $${\left\{\bar{\varepsilon },T\right\}}_{n+\alpha }=\left(1-\alpha \right){\left\{\bar{\varepsilon },T\right\}}_{n}+\alpha {\left\{\bar{\varepsilon },T\right\}}_{n+1}$$

where α∈[0,1] is an algorithmic parameter [46]. For other ways of integration, which may not exactly satisfy the second principle of thermodynamics, the interested reader is referred to [24].

### 4.3. Adjustment to the Johnson–Cook Plasticity Model

Because of its simplicity and because it is one of the most commonly used rate-dependent models, the Johnson–Cook (JC) model was used throughout this thesis to describe visco-plastic material behavior [47,48]. This model was deliberately introduced in a dedicated chapter to highlight its implications and to allow for a fairly straightforward adaptation of the overall approach to other constitutive models. In fact, an additional constitutive model will be treated in Section 5.2. Since this model will be restricted to this subsection only, an introduction in this section is deliberately omitted in order not to introduce unnecessary complexity. The JC model gives a constitutive equation that is suitable for metals subjected to large strains, high strain rates, and high temperatures, such as in [35]

(33) $${\bar{\sigma }}_{y}\left({\dot{\bar{\varepsilon }}}^{p},\bar{\varepsilon },T\right)=\left(A+B{\left({\bar{\varepsilon }}^{p}\right)}^{n}\right)\left[1+C\ln\left(\frac{{\dot{\bar{\varepsilon }}}^{p}}{{\dot{\bar{\varepsilon }}}_{0}}\right)\right]\left(1-\theta^{q}\right)$$

with the nondimensional temperature θ being defined as

(34) $$\theta =\left\{\begin{array}{cc} 0 & \mathrm{for}\;T\leq T_{r},\\ \frac{T-T_{r}}{T_{m}-T_{r}} & \mathrm{for}\;T_{r}<T\leq T_{m},\\ 1 & \mathrm{for}\;T>T_{m}. \end{array}\right.$$

Here, ${\bar{\sigma }}_{y}$ is the equivalent stress corresponding to the yield stress $\sigma_{y}$. The parameters A,B,C, *n*, and *q* are material constants; ${\dot{\varepsilon }}_{0}$ is a reference strain rate; $T_{m}$ is the melting temperature; and $T_{r}$ is the reference temperature (which is usually room temperature). The term in the first set of parentheses in Equation (33) gives a stress as a function of strain, where *B* is the strain hardening coefficient and *n* is the strain hardening exponent. The terms in the second and third sets of parentheses represent the effects of strain rate and temperature, respectively [48].

Based on Equations (7), (9), and (12)—as well as through considering the neglect of an elastic energy $W^{e}$ and the fact that the stored thermal energy $W^{th}$ and the Fourier heat conduction potential χ do not depend on strains or strain rates—it can be stated that

(35) $$\bar{\sigma }=\frac{\partial W^{p}}{\partial {\bar{\varepsilon }}^{p}}+\frac{\partial \psi *}{\partial {\dot{\bar{\varepsilon }}}^{p}}.$$

To the best of the authors’ knowledge, this relation has never been explicitly stated in the EBVM literature. When using the assumed neglect of the elastic stored energy $W^{e}$, it even contradicted what was stated by Stainier and Ortiz [27]. However, it was consistent with the terms for $W^{p}$ and ψ* used by Su and Stainier [35] (see also Su [42] (p. 78)). Furthermore, Section 5.2 will show that the stress data that were calculated using Equation (35) were in good overall agreement with the stress data reported by Stainier and Ortiz [27]. Regarding its physical interpretation, this division of the stress was considered to be in accordance with the general approach presented by Ziegler and Wehrli [49], who divided the stress into a quasi-conservative and a dissipative part. To further substantiate the similarity, the quasi-conservative stress therein resulted from a partial derivative of the free energy with respect to the strains, and the dissipative part was related to a partial derivative of a dissipation function with respect to the strain rate [49,50].

Assuming that, due to the neglect of elastic strains, the equivalent stress $\bar{\sigma }$ coincides with the equivalent yield curve ${\bar{\sigma }}_{y}$, Equation (33) leads to a possible description of the plastic stored energy potential as [35]

(36) $$W^{p}\left({\bar{\varepsilon }}^{p}\right)=\left(A_{s}{\bar{\varepsilon }}^{p}+\frac{B_{s}}{n+1}{\left({\bar{\varepsilon }}^{p}\right)}^{n+1}\right)\left(1-\theta^{q}\right)$$

and to the dissipation pseudopotential in the form of [42]

(37) $$\begin{array}{cc} \psi *\left({\dot{\bar{\varepsilon }}}^{p},{\bar{\varepsilon }}^{p},T\right) & =\left(1-\theta^{q}\right)[\left(A_{d}+B_{d}{\left({\bar{\varepsilon }}^{p}\right)}^{n}\right){\dot{\bar{\varepsilon }}}^{p}\\ \; & +\left(A+B{\left({\bar{\varepsilon }}^{p}\right)}^{n}\right)C\left({\dot{\bar{\varepsilon }}}^{p}\ln\frac{{\dot{\bar{\varepsilon }}}^{p}}{{\dot{\varepsilon }}_{0}}-{\dot{\bar{\varepsilon }}}^{p}+{\dot{\varepsilon }}_{0}\right)]. \end{array}$$

Here, as well as according to Su and Stainier [35], the parameters $A_{s},B_{s},A_{d}$, and $B_{d}$ correspond to the stored and dissipative parts of the parameters *A* and *B*. Thus, they can be, respectively, expressed as $A_{s}+A_{d}=A$ and $B_{s}+B_{d}=B$ [35]. It should be noted that, however, the strain rate-dependent part of the constitutive equation was modeled exclusively in the dissipation pseudopotential, which includes $A_{d}$ and $B_{d}$ as part of *A* and *B*, respectively. Thus, the physical implications were not as distinct as the naming suggests. Nevertheless, the relative values of these parameters have a strong influence on the distribution of the plastic work when they are converted into heat. However, their ratio is not identical to the Taylor–Quinney coefficient, as will be explained in Section 4.5.

With respect to the specific entropy introduced in Section 4.2, the above equations lead to

(38) $$\rho_{0}\eta =-\frac{\partial W}{\partial T}=\rho_{0}c_{p}\ln\frac{T}{T_{r}}+\left(A_{s}{\bar{\varepsilon }}^{p}+\frac{B_{s}}{n+1}{\left({\bar{\varepsilon }}^{p}\right)}^{n+1}\right)\frac{q}{T_{m}-T_{r}}\theta^{q-1}.$$

### 4.4. Implications of Given Displacement Fields

The given time-resolved displacement fields also contained information about strain and strain rate fields. Equation (27) already stated how to calculate the equivalent plastic strain rate ${\dot{\bar{\varepsilon }}}^{p}$. By neglecting the path dependence of the deformation, the equivalent strains could be calculated in a similar manner as

(39) $${\bar{\varepsilon }}^{p}=\sqrt{\frac{2}{3}\varepsilon^{p}\cdot \varepsilon^{p}}.$$

However, this neglect only gives a reasonable approximation for time steps Δt→0. Furthermore, a strain measure that is independent of the path can lead to negative increments $\Delta {\bar{\varepsilon }}^{p}={\bar{\varepsilon }}_{n+1}^{p}-{\bar{\varepsilon }}_{n}^{p}$, e.g., when the loading direction changes.

Considering a rather simple explicit approximation of the temperature increment in the style of Equation (1) as $\Delta T\approx \beta_{\mathrm{int}}\frac{\sigma \Delta \varepsilon }{\rho c_{p}\Delta t}$, the effect of such negative increments is particularly evident: in the illustrative example of cyclic loading, this would mean that the specimen first heats up and then cools down when the loading direction is reversed, thus tending toward the initial temperature as it approaches its initial geometric state. Mathematically, in the presented variational model, a negative equivalent strain increment can lead to imaginary components in the potentials or pseudo-potentials, which inhibit the finding of the stationary temperature field. Therefore, and despite being aware of the introduced inaccuracy, the authors recommend replacing $\frac{\Delta {\bar{\varepsilon }}^{p}}{\Delta t}$ with ${\dot{\bar{\varepsilon }}}^{p}$ in Equation (31) in cases where there are no path-dependent equivalent strains available. To improve the accuracy of the results, the equivalent strain fields used throughout this paper were calculated with respect to path dependence. For details on the methodology, the interested reader is referred to Hartmann et al. [51].

Since the displacement fields are given and ${\bar{\varepsilon }}^{p}$ is the only internal variable as stated in Section 4.2, the optimization with respect to it in Equations (23) and (24) becomes obsolete. Therefore, the optimization problem is reduced to

(40) $$T_{n+1}=\arg\;\underset{T_{n+1}}{\sup}\;\Phi_{n}\left(T_{n+1}\right)$$

with the function $\Phi_{n}$ represented as

(41) $$\Phi_{n}=\int_{\Omega }W_{n+1}-W_{n}+\rho_{0}\eta_{n}\left(T_{n+1}-T_{n}\right)+\int_{t_{n}}^{t_{n+1}}\psi *\prime dt\prime -\Delta t\chi \left(\frac{\nabla T_{n+1}}{T_{n+1}},T_{n}\right)d\Omega .$$

To bypass the optimization and allow for a direct solution of a system of nonlinear equations, the functional $\Phi_{n}$ could be derived analytically with respect to $T_{n+1}$ in future research, thus eventually leading to

(42) $$\frac{\partial \Phi_{n}\left(T_{n+1}\right)}{\partial T_{n+1}}\overset{!}{=}0.$$

### 4.5. Calculation of the Taylor–Quinney Coefficient

Based on the EBVM introduced in the previous subsections, the TQC β and factor $\beta_{\mathrm{diff}}$ can be calculated a posteriori [27]. As mentioned in Section 2, $\beta_{\mathrm{diff}}$ was the factor used in the heat equation. It is common to refer to $\beta_{\mathrm{diff}}$ as the differential form of the TQC. Strictly speaking, however, these two factors, β and $\beta_{\mathrm{diff}}$, must be distinguished in the case of a temperature-dependent $W^{p}=W^{p}\left({\bar{\varepsilon }}^{p},T\right)$. As introduced in Section 2, the TQC $\beta_{\mathrm{int}}=\beta$ is commonly used to describe the fraction of plastic work converted into heat and, for simplicity by maintaining a limited set of variables, is also used to describe the fraction of plastic work rate ${\dot{A}}^{p}$ converted into heat rate ${\dot{Q}}^{p}$ as

(43) $$\beta ={\dot{Q}}^{p}/{\dot{A}}^{p}$$

with ${\dot{A}}^{p}=\sigma {\dot{\varepsilon }}^{p}$ [4]. If $W^{p}$ decreases solely due to a change in temperature, the resulting difference $\Delta W^{p}$ also contributes to an increase in temperature. As a consequence, in the case of a complete dissipation of the currently introduced deformation work rate ${\dot{A}}^{p}$ with a simultaneous decrease in $W^{p}$ due to temperature, $\beta_{\mathrm{diff}}$ can take values of $\beta_{\mathrm{diff}}>1$. However, the fraction of the plastic work rate ${\dot{A}}^{p}$ in the heating rate ${\dot{Q}}^{p}$ cannot, by definition, be greater than one, i.e., β≯1.

It should be noted that neither Rosakis et al. [4] nor Stainier and Ortiz [27], on whose work this section is largely based, explicitly distinguished between β, $\beta_{\mathrm{int}}$, and $\beta_{\mathrm{diff}}$, but rather they made a tacit assumption. For the sake of clarity and consistency, however, this distinction in nomenclature will be maintained throughout the rest of this paper.

To derive the corresponding relation for β for the EBVM under consideration, this work follows Stainier and Ortiz [27], who provide equations for the dissipation as $D_{int}=Y{\dot{\bar{\varepsilon }}}^{p}$ and $D_{int}=\beta_{\mathrm{diff}}S\cdot D^{p}$, where *Y* is the scalar thermodynamic driving force, S is the Mandel stress tensor, and $D^{p}$ is the plastic deformation rate. Using the scalar value corresponding to the Cauchy stress tensor σ and consistently neglecting elastic strains, the latter equation can be rewritten as $D_{int}=\beta_{\mathrm{diff}}\sigma {\dot{\varepsilon }}^{p}$, or due to the work conjugate $\sigma {\dot{\varepsilon }}^{p}=\bar{\sigma }{\dot{\bar{\varepsilon }}}^{p}$ as $D_{int}=\beta_{\mathrm{diff}}\bar{\sigma }{\dot{\bar{\varepsilon }}}^{p}$. Combining the two equations gives $\beta \bar{\sigma }{\dot{\bar{\varepsilon }}}^{p}=Y{\dot{\bar{\varepsilon }}}^{p}$. Rearranging and using Equations (13) and (33) in the form $Y=\frac{\partial \psi *}{\partial {\dot{\bar{\varepsilon }}}^{p}}$ finally yields

(44) $$\beta =\frac{\frac{\partial \psi *}{\partial {\dot{\bar{\varepsilon }}}^{p}}}{\bar{\sigma }}=\frac{\frac{\partial \psi *}{\partial {\dot{\bar{\varepsilon }}}^{p}}}{\frac{\partial W^{p}}{\partial {\bar{\varepsilon }}^{p}}+\frac{\partial \psi *}{\partial {\dot{\bar{\varepsilon }}}^{p}}}.$$

By including Equation (33) and the derivative of Equation (37) with respect to ${\dot{\bar{\varepsilon }}}^{p}$, we obtain

(45) $$\beta =\beta \left({\bar{\varepsilon }}^{p},{\dot{\bar{\varepsilon }}}^{p}\right)=\frac{A_{d}+B_{d}{\left({\bar{\varepsilon }}^{p}\right)}^{n}+\left(A+B{\left({\bar{\varepsilon }}^{p}\right)}^{n}\right)C\ln\frac{{\dot{\bar{\varepsilon }}}^{p}}{\varepsilon_{0}}}{\left(A+B{\left({\bar{\varepsilon }}^{p}\right)}^{n}\right)\left(1+C\ln\frac{{\dot{\bar{\varepsilon }}}^{p}}{\varepsilon_{0}}\right)}.$$

Here it becomes obvious that β does not depend on the temperature *T*. Thus the a posteriori calculation of the TQC becomes an a priori calculation in the case of given displacement fields. As a side note, this cancellation of the temperature, when assuming a similar decomposition into $W^{p}$ and ψ* as for the JC model presented, holds for any approach that models the flow stress with a multiplicative incorporation of the temperature or any term exclusively depending on it as a variable, e.g., the model proposed by Klopp et al. [52] or the KHL constitutive models [53,54]. However, it is not generally applicable. The equation for $\beta_{\mathrm{diff}}$ is derived as

(46) $$\beta_{\mathrm{diff}}^{SO}=\frac{T\frac{\partial {\bar{\sigma }}_{W^{p}}}{\partial T}+{\bar{\sigma }}_{\psi *}}{\bar{\sigma }}$$

by Stainier and Ortiz [27] where ${\bar{\sigma }}_{W^{p}}=\frac{\partial W^{p}}{\partial {\bar{\varepsilon }}^{p}}$ and ${\bar{\sigma }}_{\psi *}=\frac{\partial \psi *}{\partial {\dot{\bar{\varepsilon }}}^{p}}$ are the respective parts of the equivalent stress $\bar{\sigma }$. The superscript ${\left(\cdot \right)}^{SO}$ refers to the authors of the respective paper and is used for distinction. It will be shown in Section 5 that the inclusion of this factor $\beta_{\mathrm{diff}}^{SO}$ in the adiabatic heat equation

(47) $$\rho_{0}c_{p}\dot{T}=\beta_{\mathrm{diff}}^{SO}{\dot{A}}^{p}=\beta_{\mathrm{diff}}^{SO}\bar{\sigma }{\dot{\bar{\varepsilon }}}^{p}$$

results in the same temperature evolution as the EBVM, at least for when the JC parameter q=1. It should be mentioned that, from an energy conservation point of view, the case of a non-linear dependence of $W^{p}$ on *T* with the simultaneous use of a Helmholtz thermal free energy $W^{th}$ leads to an additional term in the heat equation. The derivation can be found in Appendix A.1. For the presented JC version of the EBVM, this additional term vanishes for q=1, which may explain the discrepancies between the temperature resulting from the EBVM and from Equation (48) for q≠1.

With a rearrangement of Equation (44) as $\beta =1-\frac{\partial W^{p}}{\partial {\bar{\varepsilon }}^{p}}/\bar{\sigma }$, the proximity to the approach proposed by Rosakis et al. [4] becomes evident. For the common assumption that the only internal variable on which the stored plastic energy depends is the plastic strain, as well as for treating the one-dimensional case without using equivalent strains and strain rates, they proposed $\beta_{\mathrm{diff}}=1-\tilde{E}\prime \left(\varepsilon^{p}\right)/\tilde{\sigma }$, where $\tilde{E}$ is the stored cold work; $\tilde{E}\prime$ is its derivative with respect to $\varepsilon^{p}$; and $\tilde{\sigma }$ is a stress function that, for the most general case, can depend on $\varepsilon^{p},{\dot{\varepsilon }}^{p}$, and *T*.

In cases where the plastic free energy does not depend on the temperature $W^{p}\neq W^{p}\left(T\right)$, the term $T\frac{\partial {\bar{\sigma }}_{W^{p}}}{\partial T}$ in Equation (46) vanishes, thereby resulting in

(48) $$\beta_{\mathrm{diff}}^{SO}=\beta =\frac{\frac{\partial \psi *}{\partial {\dot{\bar{\varepsilon }}}^{p}}}{\bar{\sigma }}=1-\frac{\frac{\partial W^{p}}{\partial {\bar{\varepsilon }}^{p}}}{\bar{\sigma }}.$$

Thus, the most important difference is the exclusive dependence of $\tilde{E}$ on the cumulative plastic strain, which is only an assumption of Rosakis et al. [4], and this is in contrast to the dependence of $W^{p}$ on the cumulative plastic strain, as well as on the temperature, in the EBVM. The second difference is the assumption of the stress function $\tilde{\sigma }$, which is also used to model $\tilde{E}$. This function $\tilde{\sigma }$ does not coincide with the constitutive equation for the yield stress $\sigma_{y}$ [4].

In contrast, $W^{p}$ is a result of the partition of $\sigma_{y}$ (according to Equation (33)). The specific form of this split is itself an assumption, since, to the best of the authors’ knowledge, the only additional requirements are the convexity of $\psi *\left({\dot{\bar{\varepsilon }}}^{p}\right)$, as was required in Section 4.2, and the fact that—for the sake of thermodynamic consistency—the stored potential energy $W^{p}$ must not depend on the strain rate [4]. Thus, the formulation of $\beta_{\mathrm{diff}}$ derived from the EBVM in this paper appears to be more general while requiring fewer assumptions. Despite the improved consistency of the approach, it should still be carefully noted that the assumption of the explicit modeling of $\beta_{\mathrm{diff}}$ is ultimately just replaced by another assumption of the expressions for $W^{p}$ and ψ*.

Due to the lack of an illustrative physical interpretation of the definition of $\beta_{\mathrm{diff}}$ in Equations (46) and (47), a descriptive approximation $\beta_{\mathrm{diff}}^{\mathrm{approx}}$ was introduced as

(49) $$\beta_{\mathrm{diff}}^{\mathrm{approx}}=1-\frac{\Delta W^{p}}{\Delta A^{p}}\approx 1-\frac{W_{n+1}^{p}-W_{n}^{p}}{\Delta t\bar{\sigma }{\dot{\bar{\varepsilon }}}^{p}}.$$

This approximation, despite its reduced accuracy, allows a clear interpretation, i.e., the full amount of plastic deformation work in each incremental step $\Delta A^{p}$ that is not stored as plastic potential energy $W^{p}$ is dissipated as heat and thus contributes to an increase in temperature. This approximation also takes into account the exclusively temperature-dependent decrease in $W^{p}$.

## 5. Analysis of the Energy-Based Variational Model

In the following subsections, the EBVM is evaluated by comparing the resulting temperature with the reference temperatures derived from the adiabatic heat equation, i.e., the heat equation that neglects conductive heat transfer,

(50) $$\rho_{0}c_{p}\dot{T}=\beta_{\mathrm{diff}}\bar{\sigma }{\dot{\bar{\varepsilon }}}^{p}.$$

The specific formulation of the $\beta_{\mathrm{diff}}$ factors was introduced as appropriate. The implementation and all corresponding calculations were performed with The Mathworks, Inc. [55]. Furthermore, for the sake of clarity, only a single material point equipped with a synthetic data set for strains and strain rates will be considered. The corresponding integral over the domain Ω in Equation (41) therefore vanishes. It should be noted that, in general, there is no need to calculate this integral if heat conduction is neglected. When taking the displacement fields as given, the temperatures at the spatial points were coupled only by the heat conduction term χ. Without this conduction, Equation (41) could be rewritten as

(51) $$\Phi_{n}=\int_{\Omega }W\left(T_{n+1}\right)d\Omega$$

with the auxiliary function

(52) $$W\left(T_{n+1}\right)=W_{n+1}-W_{n}+\rho_{0}\eta_{n}\left(T_{n+1}-T_{n}\right)+\int_{t_{n}}^{t_{n+1}}\psi *\prime dt\prime .$$

If the integral over Ω is now approximated as a sum over *m* infinitesimally small elements, the functional $\Phi_{n}$ can be written as

(53) $$\Phi_{n}=\sum_{i=1}^{m}V_{i}\cdot W_{i}\left(T_{n+1,i}\right),$$

where $V_{i}$ is the corresponding infinitesimal volume of element *i*. Due to additivity, the temperature $T_{n+1,i}$ for each element *i* is optimized to find the stationary point of $W_{i}$ with respect to $T_{n+1,i}$. In other words, the stationary value of the integrated potential $\Phi_{n}$ is the sum of the stationary values of the subpotentials $W_{i}$ multiplied by the corresponding infinitesimal volume $V_{i}$. Since the values of $V_{i}$ have no influence, and as the total potential $\Phi_{n}$ serves only as a vehicle while its value is irrelevant, the temperatures can be obtained by local optimization as long as the conductive coupling is neglected and the strain and strain rate fields are given.

The synthetic data set mentioned above consists of equivalent plastic strains ${\bar{\varepsilon }}^{p}=\left[0,0.4\right]$ and a constant equivalent plastic strain rate ${\dot{\bar{\varepsilon }}}^{p}=1000\;s^{-1}$. The calculation uses $num_{steps}=200$ time steps and thus, assuming path independence, a corresponding time step size $\Delta t=\frac{max({\bar{\varepsilon }}^{p})}{num_{steps}\cdot {\dot{\bar{\varepsilon }}}^{p}}=1\times 10^{-6}\;s$. Furthermore, it is irrelevant whether the equivalent strain and strain rate values, ${\bar{\varepsilon }}^{p}$ and ${\dot{\bar{\varepsilon }}}^{p}$, or the true strains and strain rates, $\varepsilon^{p}$ and ${\dot{\varepsilon }}^{p}$, are used in the case of uniaxial loading. This equivalence is derived in Appendix A.2, and it is crucial for the calibration of the parameters. Although irrelevant for the evaluation, it should be noted that the assumption of adiabaticity, i.e., λ=0 or χ=0 in the case of a single material point, is indeed a valid approximation for strain rates as high as those used [6].

### 5.1. Examination with the Johnson–Cook Constitutive Model

Although the resulting temperatures will not be discussed in terms of their absolute values, this section will nevertheless use actual JC material parameters for the titanium alloy Ti-6Al-4V, as obtained in a prominent paper by Lee and Lin [48]. In addition to the parameters A=724.7 MPa, B=683.1 MPa, C=0.035, n=0.47, q=1.0, and ${\dot{\varepsilon }}_{0}=1\times 10^{-5}$ $s^{-1}$, a mass density $\rho_{0}=4430\;\mathrm{kg}\;m^{-3}$ was used and the specific heat capacity $c_{p}$ was converted from $0.13\;\mathrm{cal}{\left(g\;{}^{\circ}C\right)}^{-1}$ to $518\;{\mathrm{Jkg}}^{-1}K^{-1}$. Since Lee and Lin [48] did not explicitly specify the melting temperature $T_{m}$—i.e., the actual melting temperature of Ti-6Al-4V—$T_{m}=1604$ °C was used [56].

As indicated in Section 4.3, splitting the parameters *A* and *B* into $A_{s},A_{d}$ and $B_{s},B_{d}$ had a significant effect on the fraction of plastic work converted into heat, although it was found to be neither identical to the TQC β nor to the factor $\beta_{\mathrm{diff}}$. To illustrate the justification of the EBVM, a split factor was introduced as $\beta_{\mathrm{split}}=\frac{A_{d}}{A}=\frac{B_{d}}{B}$. The actual values of this split and the fact that the split was identical for *A* and *B* were arbitrary in nature and only enhanced the comprehensibility of the overall evaluation.

For this evaluation, four different temperature histories were calculated: (1) $T^{\left(a\right)}$, which results from the EBVM by solving Equation (40); (2) $T^{\left(b\right)}$, which results from using the factor $\beta_{\mathrm{diff}}^{SO}$, as in Equation (46), in the adiabatic heat Equation (50); (3) $T^{\left(c\right)}$, which results from a similar use of β, as defined in Equations (44) and (45); (4) $T^{\left(d\right)}$, which is obtained from $\beta_{\mathrm{diff}}^{\mathrm{approx}}$, as in Equation (49). The superscripts ${\left(\cdot \right)}^{\left(\cdot \right)}$ are retained for clarity. The exact calculation for the temperature evolution $T^{\left(b\right)}$ to $T^{\left(d\right)}$ will be explained in the following paragraphs.

The evolution of $T^{\left(b\right)}$ was determined based on $\beta_{\mathrm{diff}}^{SO}=\beta_{\mathrm{diff}}^{SO}\left(T^{\left(a\right)},{\bar{\varepsilon }}^{p},{\dot{\bar{\varepsilon }}}^{p}\right)$ using $T^{\left(a\right)}$ as an input to accurately describe the $\beta_{\mathrm{diff}}$ factor resulting from the EBVM. For each incremental step, the temperature $T_{n+1}^{\left(b\right)}$ was then calculated as

(54) $$T_{n+1}^{\left(b\right)}=T_{n}^{\left(b\right)}+\beta_{\mathrm{diff}}^{SO}\left(T_{n+1}^{\left(a\right)},{\bar{\varepsilon }}_{n+1}^{p},{\dot{\bar{\varepsilon }}}_{n+1}^{p}\right)\frac{{\bar{\sigma }}^{\left(b\right)}\left(T_{n}^{\left(b\right)},{\bar{\varepsilon }}_{n+1}^{p},{\dot{\bar{\varepsilon }}}_{n+1}^{p}\right){\dot{\bar{\varepsilon }}}_{n+1}^{p}\Delta t}{\rho_{0}c_{p}}.$$

The use of ${\bar{\sigma }}^{\left(b\right)}\left(T^{\left(b\right)},{\bar{\varepsilon }}^{p},{\dot{\bar{\varepsilon }}}^{p}\right)$ instead of ${\bar{\sigma }}^{\left(a\right)}\left(T^{\left(a\right)},{\bar{\varepsilon }}^{p},{\dot{\bar{\varepsilon }}}^{p}\right)$ may be controversial. Aside from not making a significant difference, this choice was made to correctly describe the current plastic work rate ${\dot{A}}^{p}$ as a function of the current stress σ, which—in turn—depends on the current temperature *T* and could potentially show a discrepancy between $T^{\left(a\right)}$ and $T^{\left(b\right)}$.

The choice of which indices, *n* or n+1, to use to calculate $T_{n+1}$ is a matter of the time integration method used. This topic is treated in more detail in Section 6, but suffice it to say here that the calculation as shown above, but using ${\bar{\sigma }}^{\left(b\right)}\left(T_{n+1}^{\left(b\right)},{\bar{\varepsilon }}_{n+1}^{p},{\dot{\bar{\varepsilon }}}_{n+1}^{p}\right)$ instead, would be the usual implicit or backward Euler method, thus introducing a nonlinearity. Equation (54) is therefore only an approximation, with the results being between those for the explicit and implicit Euler schemes. However, due to the small synthetic time steps used, the choice of time integration method was insignificant but the authors considered this choice to be the most illustrative. Recalling Equations (1) and (2), it becomes apparent that Equation (54) uses $\beta_{\mathrm{diff}}$ instead of $\beta_{\mathrm{int}}$ in combination with $\Delta A^{p}=\Delta t\bar{\sigma }{\dot{\bar{\varepsilon }}}^{p}$. This is due to the fact that $\Delta A^{p}/\Delta t$ refers to a time step that is deliberately small enough to approximate the rate $\Delta A^{p}/\Delta t\approx {\dot{A}}^{p}$.

To determine $T^{\left(c\right)}$, β is calculated a priori from the strain and strain rate data (as in Equation (45)), which is followed by a stepwise approximate solution of the adiabatic heat Equation (50). For this approximate solution, a similar time integration method was used as for the determination of $T^{\left(b\right)}$, which finally led to

(55) $$T_{n+1}^{\left(c\right)}=T_{n}^{\left(c\right)}+\beta \left({\bar{\varepsilon }}_{n+1}^{p},{\dot{\bar{\varepsilon }}}_{n+1}^{p}\right)\frac{{\bar{\sigma }}^{\left(c\right)}\left(T_{n}^{\left(c\right)},{\bar{\varepsilon }}_{n+1}^{p},{\dot{\bar{\varepsilon }}}_{n+1}^{p}\right){\dot{\bar{\varepsilon }}}_{n+1}^{p}\Delta t}{\rho_{0}c_{p}}.$$

$T^{\left(d\right)}$ is calculated based on $\beta_{\mathrm{diff}}^{\mathrm{approx}}$ as defined in Equation (49). Like $\beta_{\mathrm{diff}}^{SO}$, $\beta_{\mathrm{diff}}^{\mathrm{approx}}$ depends on the state variables of the EBVM and is therefore calculated a posteriori as

(56) $$\beta_{\mathrm{diff},n+1}^{\mathrm{approx}}=1-\frac{W_{n+1}^{p}\left(T_{n+1}^{\left(a\right)}\right)-W_{n}^{p}\left(T_{n}^{\left(a\right)}\right)}{\Delta t{\bar{\sigma }}^{\left(a\right)}\left(T_{n+1}^{\left(a\right)},{\bar{\varepsilon }}_{n+1}^{p},{\dot{\bar{\varepsilon }}}_{n+1}^{p}\right){\dot{\bar{\varepsilon }}}_{n+1}^{p}}.$$

The temperature $T_{n+1}^{\left(d\right)}$ is then calculated as

(57) $$T_{n+1}^{\left(d\right)}=T_{n}^{\left(d\right)}+\beta_{\mathrm{diff},n+1}^{\mathrm{approx}}\frac{{\bar{\sigma }}^{\left(d\right)}\left(T_{n}^{\left(d\right)},{\bar{\varepsilon }}_{n+1}^{p},{\dot{\bar{\varepsilon }}}_{n+1}^{p}\right){\dot{\bar{\varepsilon }}}_{n+1}^{p}\Delta t}{\rho_{0}c_{p}}.$$

**Figure 1:**
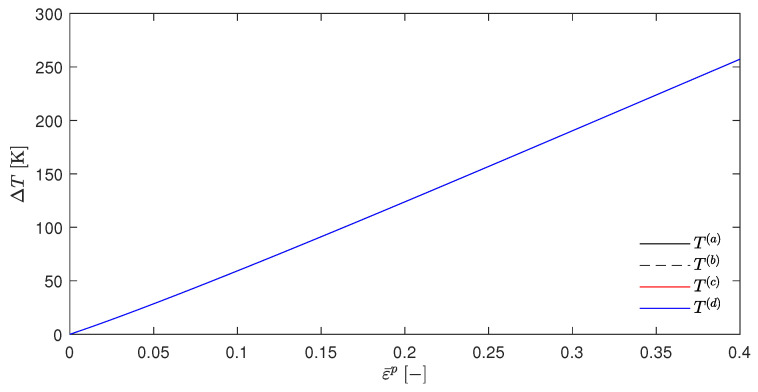
Temperature rise for $T^{\left(a\right)}$ (EBVM), $T^{\left(b\right)}$ ($\beta_{\mathrm{diff}}^{SO}$), $T^{\left(c\right)}$ (β) and $T^{\left(d\right)}$ ($\beta_{\mathrm{diff}}^{\mathrm{approx}}$) with $\beta_{\mathrm{split}}=1$.

As for the calculation of $T^{\left(b\right)}$, it is again controversial to replace ${\bar{\sigma }}^{\left(d\right)}$ with ${\bar{\sigma }}^{\left(a\right)}$. However, the above argument in favor of ${\bar{\sigma }}^{\left(d\right)}$ is similarly valid for this case. Plotting the evolution of the temperature rise $\Delta T=T-T_{0}$ for the calculated temperatures $T^{\left(a\right)}$ to $T^{\left(d\right)}$ over the equivalent strains ${\bar{\varepsilon }}^{p}$, yields the Figure 1 for $\beta_{\mathrm{split}}=1$.

It can be clearly observed that all four temperatures were nearly congruent. The scenario where no plastic Helmholtz free energy is stored, i.e., $W^{p}=0$, was also investigated by Yang et al. [24], Su [42] and Su and Stainier [35].

**Figure 2:**
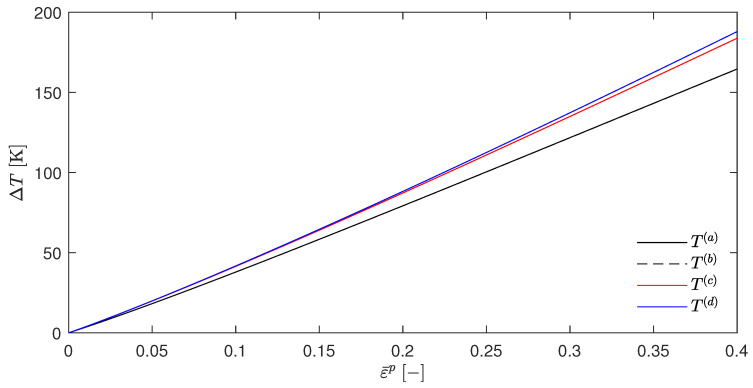
Temperature rise for $T^{\left(a\right)}$ (EBVM), $T^{\left(b\right)}$ ($\beta_{\mathrm{diff}}^{SO}$), $T^{\left(c\right)}$ (β), and $T^{\left(d\right)}$ ($\beta_{\mathrm{diff}}^{\mathrm{approx}}$) with $\beta_{\mathrm{split}}=0.5$.

**Figure 3:**
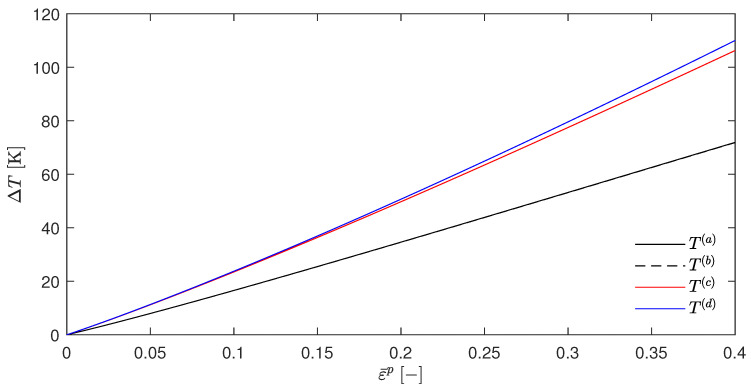
Temperature rise for $T^{\left(a\right)}$ (EBVM), $T^{\left(b\right)}$ ($\beta_{\mathrm{diff}}^{SO}$), $T^{\left(c\right)}$ (β), and $T^{\left(d\right)}$ ($\beta_{\mathrm{diff}}^{\mathrm{approx}}$) with $\beta_{\mathrm{split}}=0$.

Choosing, e.g., $\beta_{\mathrm{split}}=0.5$ gives the Figure 2. It can be seen that the temperature rises for $T^{\left(a\right)}$ (which resulted from the EBVM) and $T^{\left(b\right)}$ (which resulted from a calculation with $\beta_{\mathrm{diff}}^{SO}$) were nearly identical, as was already predicted in Section 4.5. However, the temperature evolution of $T^{\left(c\right)}$ and $T^{\left(d\right)}$ were significantly different. Another interesting feature was the close proximity of these two curves. The choice of $\beta_{\mathrm{split}}=0$, as shown in Figure 3, further emphasizes these qualitative observations.

This has two important implications. First, the congruence of $T^{\left(a\right)}$ and $T^{\left(b\right)}$ suggests the correctness of the adjustments made to the original EBVM throughout this work and the implementation, or at least its consistency, since the EBVM yielded the results predicted by Stainier and Ortiz [27] with their definition of $\beta_{\mathrm{diff}}^{SO}$. Second, the adapted EBVM exhibited incorrectness, as $T^{\left(a\right)}$ and $T^{\left(b\right)}$ differ significantly from $T^{\left(d\right)}$. The latter was, indeed, only an approximation, but with an undeniable claim to approximate correctness based on the physically motivated derivation of the underlying $\beta_{\mathrm{diff}}^{\mathrm{approx}}$ in Equation (49). These problems of the EBVM are, by the way, in their general form and are a order of magnitude independent of a myriad of small modifications. A brief overview of the variations examined without significant impact can be found in Appendix A.3. In addition, Appendix A.4 contains the corresponding plots of the β factors, and Appendix A.5 contains a plot for the JC parameter q≠1, which—in this case—shows the predicted discrepancy between $T^{\left(a\right)}$ and $T^{\left(b\right)}$.

To put the term “incorrectness” into perspective, it should be noted that the temperature evolution obtained by the EBVM is not necessarily wrong if the model is calibrated accordingly. However, this would still imply a lack of physical interpretability if the postulated a posteriori determination of the TQC and the factor $\beta_{\mathrm{diff}}$ were not consistent with the calculated stored plastic energy $W^{p}$.

The above observations together with the theoretical considerations in Section 4.5 suggest that the discrepancies and deficiencies of the EBVM are related to a temperature-dependent $W^{p}$, i.e., for the EBVM with the JC model and the current split to $W^{p}$ and ψ* if $\beta_{\mathrm{split}}\neq 1$. Despite the congruence of $T^{\left(a\right)}$ and $T^{\left(b\right)}$, the errors in the adaptation regarding the case of given displacement fields and neglected elastic strains (or in the implementation) cannot be excluded. Therefore, in the following section, the EBVM will be further evaluated with another constitutive model.

### 5.2. Examination with the Stainier–Ortiz Constitutive Model

This constitutive model was deliberately not introduced in Section 4, since it will be used exclusively in this section and a double assignment of the variables would have introduced an avoidable additional complexity into the matter. The model is taken from a paper by Stainier and Ortiz [27] and will therefore be referred to as the Stainier–Ortiz (SO) model. Since this model is entirely based on their work, it has been deliberately omitted to provide the same reference for each individual equation, see [27]. In contrast to the original model, the SO model in this work again omits the elastic strains $\varepsilon^{e}$ and thus also the elastic Helmholtz free energy $W^{e}$. The plastic stored energy $W^{p}$ in this model is composed of a power law term and an exponential saturation term as

(58) $$W^{p}\left({\bar{\varepsilon }}^{p},T\right)=\frac{n}{n+1}\frac{\sigma_{0}\left(T\right)}{b}{\left(1+b{\bar{\varepsilon }}^{p}\right)}^{\frac{1}{n}+1}+{\hat{\sigma }}_{0}\left(T\right)\left[{\bar{\varepsilon }}^{p}+\frac{1}{d}\exp\left(-d{\bar{\varepsilon }}^{p}\right)\right].$$

The dual dissipation pseudopotential is composed of a rate-independent term, i.e., it is homogeneous of order 1 in ${\dot{\bar{\varepsilon }}}^{p}$, and a rate-dependent power-law term as

(59) $$\psi *\left({\dot{\bar{\varepsilon }}}^{p},{\bar{\varepsilon }}^{p},T\right)={\hat{\sigma }}_{y}\left({\bar{\varepsilon }}^{p},T\right){\dot{\bar{\varepsilon }}}^{p}+\frac{m}{m+1}\sigma_{\nu }\left(T\right){\dot{\varepsilon }}_{0}{\left(\frac{{\dot{\bar{\varepsilon }}}^{p}}{{\dot{\varepsilon }}_{0}}\right)}^{\frac{1}{m}+1}.$$

The rate-independent term itself is, like $W^{p}$, composed of a power–law term and an exponential saturation term as

(60) $$\begin{array}{cc} {\hat{\sigma }}_{y}\left({\bar{\varepsilon }}^{p},T\right) & =\sigma_{1}\left(T\right){\left(1+b{\bar{\varepsilon }}^{p}\right)}^{\frac{1}{n}}+{\hat{\sigma }}_{1}\left(T\right)\left[1-\exp\left(-d\prime {\bar{\varepsilon }}^{p}\right)\right]. \end{array}$$

Using Equation (35) gives an equation for the yield stress as

(61) $$\begin{array}{cc} \sigma_{y}\left({\dot{\bar{\varepsilon }}}^{p},{\bar{\varepsilon }}^{p},T\right) & =\sigma \left({\dot{\bar{\varepsilon }}}^{p},{\bar{\varepsilon }}^{p},T\right)=\frac{\partial W^{p}}{\partial {\bar{\varepsilon }}^{p}}+\frac{\partial \psi *}{\partial {\dot{\bar{\varepsilon }}}^{p}}\\ \; & ={\hat{\sigma }}_{y}\left({\bar{\varepsilon }}^{p},T\right)+\sigma_{\nu }\left(T\right){\left(\frac{{\dot{\bar{\varepsilon }}}^{p}}{{\dot{\varepsilon }}_{0}}\right)}^{\frac{1}{m}}+\sigma_{0}\left(T\right){\left(1+b{\bar{\varepsilon }}^{p}\right)}^{\frac{1}{n}}+{\hat{\sigma }}_{0}\left(T\right)\left(1-\exp\left(-d{\bar{\varepsilon }}^{p}\right)\right). \end{array}$$

Entropy can be derived as

(62) $$\rho_{0}\eta =-\frac{\partial W}{\partial T}=\rho_{0}c_{p}\ln\frac{T}{T_{0}}+\frac{n}{n+1}\frac{\sigma_{0}\left(T_{0}\right)\omega_{0}}{b}{\left(1+b{\bar{\varepsilon }}^{p}\right)}^{\frac{1}{n}+1}+{\hat{\sigma }}_{0}\left(T_{0}\right){\hat{\omega }}_{0}\left[{\bar{\varepsilon }}^{p}+\frac{1}{d}\exp\left(-d{\bar{\varepsilon }}^{p}\right)\right]$$

and the TQC β has the form

(63) $$\beta =\frac{\frac{\partial \psi *}{\partial {\dot{\bar{\varepsilon }}}^{p}}}{\sigma }=\frac{{\hat{\sigma }}_{y}\left({\bar{\varepsilon }}^{p},T\right)+\sigma_{\nu }\left(T\right){\left(\frac{{\dot{\bar{\varepsilon }}}^{p}}{{\dot{\varepsilon }}_{0}}\right)}^{\frac{1}{m}}}{{\hat{\sigma }}_{y}\left({\bar{\varepsilon }}^{p},T\right)+\sigma_{\nu }\left(T\right){\left(\frac{{\dot{\bar{\varepsilon }}}^{p}}{{\dot{\varepsilon }}_{0}}\right)}^{\frac{1}{m}}+\sigma_{0}\left(T\right){\left(1+b{\bar{\varepsilon }}^{p}\right)}^{\frac{1}{n}}+{\hat{\sigma }}_{0}\left(T\right)\left(1-\exp\left(-d{\bar{\varepsilon }}^{p}\right)\right)}.$$

The critical stresses in the above equations are defined as

(64) $$\begin{array}{cc} \sigma_{0}\left(T\right) & =\sigma_{0}\left(T_{0}\right)\left[1-\omega_{0}\left(T-T_{0}\right)\right], \end{array}$$

(65) $$\begin{array}{cc} {\hat{\sigma }}_{0}\left(T\right) & ={\hat{\sigma }}_{0}\left(T_{0}\right)\left[1-{\hat{\omega }}_{0}\left(T-T_{0}\right)\right], \end{array}$$

(66) $$\begin{array}{cc} \sigma_{1}\left(T\right) & =\sigma_{1}\left(T_{0}\right)\left[1-\omega_{1}\left(T-T_{0}\right)\right], \end{array}$$

(67) $$\begin{array}{cc} {\hat{\sigma }}_{1}\left(T\right) & ={\hat{\sigma }}_{1}\left(T_{0}\right)\left[1-{\hat{\omega }}_{1}\left(T-T_{0}\right)\right], \end{array}$$

(68) $$\begin{array}{cc} \sigma_{\nu }\left(T\right) & =\sigma_{\nu }\left(T_{0}\right)\left[1-\omega_{\nu }\left(T-T_{0}\right)\right]. \end{array}$$

To ensure good comparability, the nomenclature utilized was identical to that used by Stainier and Ortiz [27] (except for the use of ${\hat{\sigma }}_{y}$ instead of $\sigma_{y}$ to avoid overlapping with the yield stress). The assignment of other variables, e.g., *n* (which was also used in the JC model), should remain sufficiently clear since the use of the SO model is limited to this section. It was also pointed out that all σ variables should be written as $\bar{\sigma }$ when calculating and calibrating their values with equivalent strains and strain rates in the case of a fully consistent approach regarding the nomenclature. Since the true strain σ is interchangeable with the equivalent true strain $\bar{\sigma }$ in the case of uniaxial loading (see Appendix A.2), this correction has been omitted.

Table 1 lists the SO parameters that were used by Stainier and Ortiz [27] for the aluminum alloy 2023-T3 with an initial and reference temperature $T_{0}=293\;K$, a specific heat capacity $c_{p}=875.0\;{\mathrm{Jkg}}^{-1}K^{-1}$, and a mass density $\rho_{0}=2780.0\;\mathrm{kg}\;m^{-3}$. The reduced number of variables in the Table 1 was due to the rate-independent modeling of the material behavior of the aluminum alloy.

**Figure 4:**
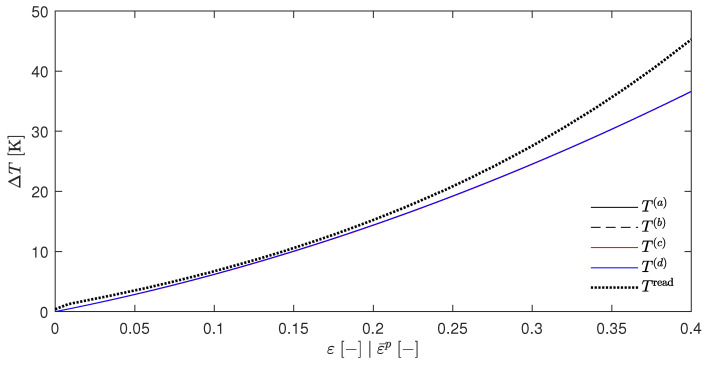
Temperature rise for $T^{\left(a\right)}$ (EBVM), $T^{\left(b\right)}$ ($\beta_{\mathrm{diff}}^{SO}$), $T^{\left(c\right)}$ (β), $T^{\left(d\right)}$ ($\beta_{\mathrm{diff}}^{\mathrm{approx}}$), and $T^{\mathrm{read}}$ extracted from the work of Stainier and Ortiz [27] for 2023-T3 aluminum alloy [27].

**Figure 5:**
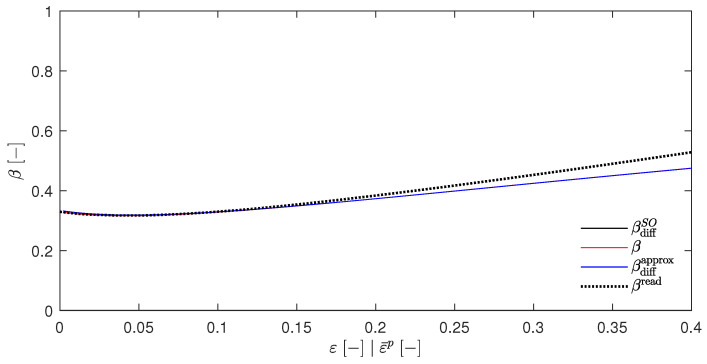
The β factors $\beta_{\mathrm{diff}}^{SO}$, β, $\beta_{\mathrm{diff}}^{\mathrm{approx}}$, and $\beta^{\mathrm{read}}$, which were extracted from the work of Stainier and Ortiz [27] for 2023-T3 aluminum alloy [27].

**Figure 6:**
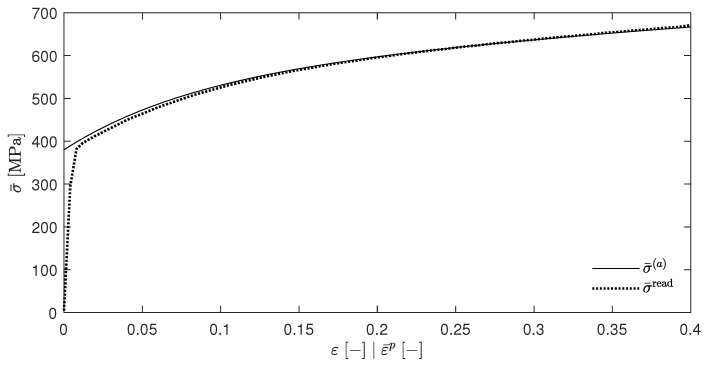
Equivalent stress $\bar{\sigma }$ and $\sigma^{\mathrm{read}}$ extracted from the work of Stainier and Ortiz [27] for 2023-T3 aluminum alloy [27].

Figure 4 shows the resulting temperature increases ΔT for the temperatures $T^{\left(a\right)}$ to $T^{\left(d\right)}$, which was calculated in the same way as in Section 5.1. Again, these curves were found to be nearly congruent, which is consistent with the above assumptions since $W^{p}$ is independent of the temperature for the material parameters that are presented in Table 1. In addition, Figure 4 also shows the temperature increase that were calculated by Stainier and Ortiz [27] as a dotted line. Furthermore, Figure 5 shows the evolution of the β-factors and Figure 6 the evolution of the stress, which were calculated using Equation (61). These two figures also include the evolution of the state variables that was reported by Stainier and Ortiz [27].

The discrepancies between the calculated and reported values of the variables may have several causes. As can be seen in particular in Figure 6, the elastic strain range was not excluded but only ignored due to its limited extent and the fact that these graphs were only for illustrative purposes. Furthermore, the exact calculation method was explicitly and deliberately not specified by Stainier and Ortiz [27]. This did not only include the time step Δt or the specific approach of evaluating the integral $\int_{t_{n}}^{t_{n+1}}\psi *\prime dt\prime$, as was the approach in Equation (31), which was introduced by Stainier [46] only one year later. Their results cannot be obtained at all by the incremental approach of the EBVM since the calculation of $W^{p}$ with the given SO material parameters for tantalum, which is also part of their research, would imply an impermissible division by zero.

Nevertheless, the calculated results show a relatively good agreement with the evolution of the state variables extracted from the work of Stainier and Ortiz [27] in all Figure 4, Figure 5 and Figure 6. This reinforces the assumption of a correct adjustment of the EBVM in Section 4, as well as a correct corresponding implementation. In particular, the good agreement of the calculated stress values seems to validate Equation (35).

**Figure 7:**
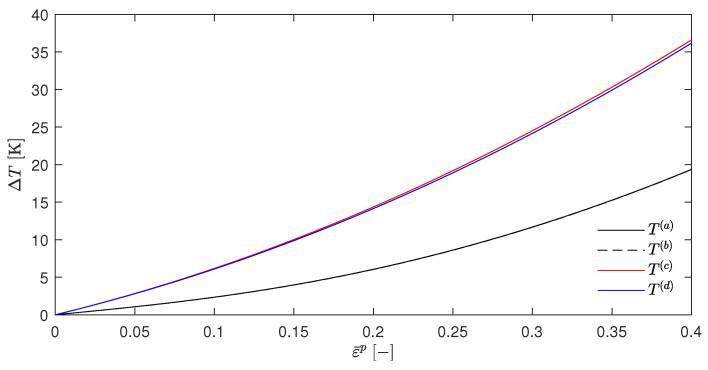
Temperature rise for $T^{\left(a\right)}$ (EBVM), $T^{\left(b\right)}$ ($\beta_{\mathrm{diff}}^{SO}$), $T^{\left(c\right)}$ (β), and $T^{\left(d\right)}$ ($\beta_{\mathrm{diff}}^{\mathrm{approx}}$) for 2023-T3 aluminum alloy with $\omega_{0}={\hat{\omega }}_{0}=0.001$.

**Figure 8:**
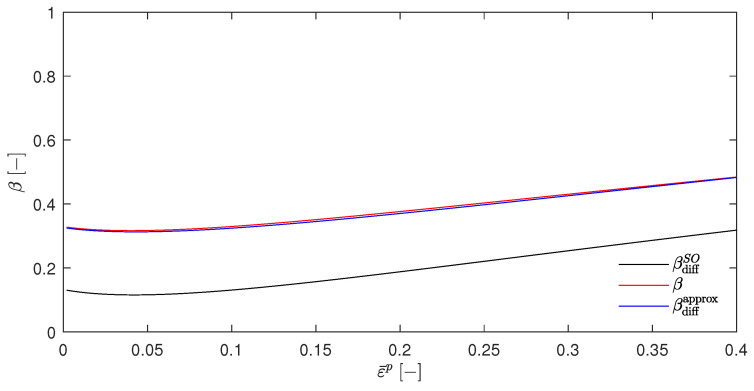
β-factors $\beta_{\mathrm{diff}}^{SO}$, β, and $\beta_{\mathrm{diff}}^{\mathrm{approx}}$ for 2023-T3 aluminum alloy with $\omega_{0}={\hat{\omega }}_{0}=0.001$.

However, if the stored plastic energy $W^{p}$ becomes temperature-dependent—e.g., by arbitrarily choosing $\omega_{0}={\hat{\omega }}_{0}=0.001$, as shown in Figure 7 and Figure 8 with the corresponding temperature and β factor evolution)—again a discrepancy between the temperatures $T^{\left(a\right)}$ (which results from the EBVM) and $T^{\left(d\right)}$ (which results from $\beta_{\mathrm{diff}}^{\mathrm{approx}}$) becomes evident. Also, a similar proximity of $T^{\left(c\right)}$ and $T^{\left(d\right)}$, as already noted in Section 5.1, can be observed.

To further substantiate the assumption of a correct adaptation and implementation, Appendix A.6 contains similar plots for temperature, β factors, and stress for α titanium—a material that was also studied by Stainier and Ortiz [27]. A comparison for tantalum is not possible, as mentioned above, because the incremental version of the EBVM does not work for the listed set of parameters.

In conclusion, the considerations of this section suggest a correct adaptation of the EBVM to given displacement fields, as well as to neglect elastic strains, the corresponding elastic Helmholtz free energy, and a correct implementation. However, these potential sources of error cannot be completely ruled out. More likely, however, is the thermodynamically inconsistent modeling of material behavior by the EBVM in the case of a temperature-dependent plastic stored energy $W^{p}$. A closed theoretical derivation of the source of the discrepancy was not possible within the scope of this work due to the limited time available. However, these potential sources of error cannot be completely ruled out. More likely, however, is the thermodynamically inconsistent modeling of material behavior by the EBVM in the case of a temperature-dependent plastic stored energy $W^{p}$. A closed theoretical derivation of the source of the discrepancy was not possible within the scope of this work due to the limited time available. However, this is a crucial step for further research. Possible starting points might be a reconsideration of the fundamental thermodynamic admissibility of a temperature-dependent plastic stored energy $W^{p}$ or the definition of stress as $\bar{\sigma }=\frac{\partial W^{p}}{\partial {\bar{\varepsilon }}^{p}}+\frac{\partial \psi *}{\partial {\dot{\bar{\varepsilon }}}^{p}}$, as shown in Equation (35). Regarding the stress definition, Ziegler and Wehrli [49], who followed a similar division of the stress into a quasi-conservative part and a dissipative part, used an additional scalar factor multiplied by the derivative of the dissipation function with respect to the strain rate to ensure an orthogonality condition. However, its implications and the transfer to the EBVM under consideration were beyond the scope of this work [49,50]. It has also been pointed out that there is a similar, but different, splitting of the stress into viscous and plastic stresses, as proposed by [57]. As they also introduced this split for the JC model, this could be a promising starting point for further research.

When considering how to determine the temperature evolution from displacement field data, two fundamentally different approaches can be followed. First, a temperature independent $W^{p}=W^{p}\left({\bar{\varepsilon }}^{p}\right)$ can be defined. This implies that, by the way (due to the modified splitting of $W^{p}$ and ψ*, which is still coupled by Equation (35)), the term $T\frac{\partial {\bar{\sigma }}_{W^{p}}}{\partial T}$ in Equation (46) vanishes. However, the temperature-dependent term in β in Equation (45) no longer vanishes. This prevents an a priori determination of $\beta_{\mathrm{diff}}$ for the case of given displacement fields. This choice of $W^{p}=W^{p}\left({\bar{\varepsilon }}^{p}\right)$ raises concerns about the calibration process. Ideally, such a calibration would be based on stress–strain curves with corresponding temperature–strain curves for the same experiments. If a temperature–strain curve was available, the direct optimization of the material parameters would be possible if the optimization with respect to temperature is replaced by an explicit analytical derivative, as in Equation (42). However, such simultaneously measured temperature–strain curves are rare. If only stress–strain curves are given, the stress and temperature over the entire deformation range must be determined iteratively with the incremental EBVM, which is then followed by a subsequent calculation of the residuals to the given stress–strain curves. This calculation process would have to be performed for all parameter combinations within an expected range, e.g., starting within [0,1500]MPa for $A_{s}$. Then, the range should be strategically narrowed, e.g., with a classical bisectional approach. These concerns, which were formulated for illustrative purposes for the special case of $W^{p}\neq W^{p}\left(T\right)$, were also applicable to the unrestricted general case of the EBVM. In order to be able to use classical optimization approaches for the constitutive parameters, i.e., eventually fitting a curve to the stress–strain curves, one has to make the assumption that, for $W^{p}\left({\bar{\varepsilon }}^{p}\right)$, the results of the EBVM are congruent with the results of the heat equation when using $\beta_{\mathrm{diff}}=1-\frac{\partial W^{p}}{\partial {\bar{\varepsilon }}^{p}}/\bar{\sigma }$ with sufficient accuracy. With this assumption, which is supported by the above considerations, the temperature evolution can be predicted explicitly and is a priori-based on stress–strain curves, thus allowing for an efficient determination of the constitutive parameters. However, in recalling Section 4.5, this special case is identical in its result to the approach proposed by [4,27]. This implies the need to justify the practical applicability of the EBVM in its current form with respect to the computational effort involved. This is a particularly crucial consideration, since its attractive advantage of modularity, i.e., the simple extension by arbitrary potentials to incorporate further physical effects (e.g., an extension by a damage term), is not used in this work again due to a limited prescribed processing time.

The second basic approach would be to assume that $\beta_{\mathrm{diff}}=\beta =\frac{\partial \psi *}{\partial {\dot{\bar{\varepsilon }}}^{p}}/\bar{\sigma }$ and obtain the temperature evolution from solving the heat equation. This assumption, $\beta_{\mathrm{diff}}=\beta$, is not theoretically motivated by thermodynamics but rather empirically motivated by the already good agreement with the temperatures obtained by using $\beta_{\mathrm{diff}}^{\mathrm{approx}}$. It can be seen as an initially arbitrary choice of a modeling approach for $\beta_{\mathrm{diff}}=\beta_{\mathrm{diff}}\left({\bar{\varepsilon }}^{p},{\dot{\bar{\varepsilon }}}^{p}\right)$ without any inherent claim to physical interpretability. With an appropriate calibration, however, it is considered to sufficiently describe the constitutional behavior. Despite a temperature-independent $\beta_{\mathrm{diff}}$, the heat equation with a consideration of heat transfer by conduction [4]

(69) $$\rho_{0}c_{p}\dot{T}-\nabla \cdot \left(\lambda \nabla T\right)=\beta_{\mathrm{diff}}{\dot{A}}^{p}=\beta_{\mathrm{diff}}\bar{\sigma }{\dot{\bar{\varepsilon }}}^{p}$$

is still a nonlinear partial differential equation. This is because, for the sake of consistency, the determination of $T_{n+1}$, ${\bar{\sigma }}_{n+1}=\bar{\sigma }\left({\bar{\varepsilon }}_{n+1}^{p},{\dot{\bar{\varepsilon }}}_{n+1}^{p},T_{n+1}\right)$ with its nonlinear dependence on $T_{n+1}$ must also be used. Nevertheless, the author assumes that the implementation of this nonlinear partial differential equation, or even a corresponding linearization, could be associated with a significant reduction in computational cost for the problem under consideration when compared to the EBVM. Considering this estimated computational advantage, the authors also strongly recommend to evaluate the overall justification of the practical applicability of the energy-based variational model in terms of the trade-off between the increased accuracy of the results by simultaneous solution of the displacement and temperature fields and the computational effort involved. Even for the general case of non-given displacement fields and a temperature-dependent $\beta_{\mathrm{diff}}$, the results could be approximated by successive evaluation: first, by solving a more classical purely mechanical problem for the displacement field, and then by a purely thermal problem for the temperature evolution. When evaluating the proof of applicability, it should be recapitulated that even the EBVM presented above is a mere approximation due to its incremental character.

Considering the improved suitability for calibration without an additional, though probably valid, assumption (and especially a computational advantage), the modeling of $\beta_{\mathrm{diff}}=\beta =\frac{\partial \psi *}{\partial {\dot{\bar{\varepsilon }}}^{p}}/\bar{\sigma }$ with a corresponding solution of heat Equation (69) will serve as the basic idea of the alternative approach for the determination of the temperature evolution shown in Section 6.

## 6. Alternative Approach

As motivated in Section 5, the alternative approach presented here is based on the assumption that $\beta_{\mathrm{diff}}=\beta =\frac{\partial \psi *}{\partial {\dot{\bar{\varepsilon }}}^{p}}/\bar{\sigma }$. With this assumption, the partial differential heat equation

(70) $$\rho_{0}c_{p}\dot{T}-\nabla \cdot \left(\lambda \nabla T\right)=\beta_{\mathrm{diff}}\bar{\sigma }{\dot{\bar{\varepsilon }}}^{p}$$

is solved using the finite element method (FEM). Furthermore, it is simplified that the stress $\bar{\sigma }$ always depends on the temperature of the previous time step $T_{n}$, no matter for which step, either *n* or n+1, $\bar{\sigma }$ is evaluated. This approximation eliminates the nonlinearity introduced by $\bar{\sigma }=\bar{\sigma }\left({\bar{\varepsilon }}^{p},{\dot{\bar{\varepsilon }}}^{p},T\right)$ in the general case of q≠1 for the JC model used. In future research, this assumption should obviously be omitted, perhaps accompanied by an appropriate linearization to ensure computational efficiency.

It should also be noted that both the thermal conductivity λ and the specific heat capacity $c_{p}$ were modeled as a function of temperature *T* in the actual calculations. Despite this temperature dependence, both were treated as constants in previous sections when calculating the derivatives with respect to temperature *T*. This simplification should have only a small impact on the resulting temperature fields, but it effectively avoids an unreasonably high level of complexity. To further minimize the impact and reduce the computational cost, both variables λ and $c_{p}$, as well as $\bar{\sigma }$, are were inserted into the respective equations for the calculation of the temperature increment with their values at the previous time step, i.e., $T_{n+1}=f\left(\lambda_{n},c_{p,n}\right)$.

For the sake of transparency and reproducibility, this section contains a brief derivation of the implemented finite element approach. Since FEM is one of the most widely used methods for solving engineering problems, its basic approach and mathematical foundations are assumed to be well known. Therefore, the derivation is deliberately short and not every single step is provided with a corresponding reference to the literature. In case of uncertainties, the interested reader is referred to the standard literature, such as the books by Belytschko et al. [58] or Zienkiewicz et al. [59]. In general, the basic approach of FEM is to divide the whole system into smaller, simpler parts: the so-called finite elements. The necessary discretization in space and, eventually, in time is already given with the displacement fields as input data. Incorporating the shape functions into the governing equations leads to a system of algebraic equations for the elements, which in turn leads to a system of equations for the entire problem.

To allow a discretization of heat Equation (70), it is necessary to integrate this equation over the respective domain, which is achieved by multiplying it by arbitrary weighting functions $v\in V=\{v\left(x,t\right)\;|\;v\in C^{1}\left(x\right)\}$ to ensure its validity for every single point in the domain Ω as

(71) $$\int_{\Omega }v\rho_{0}c_{p}\frac{\partial T}{\partial t}dx-\int_{\Omega }v\lambda \nabla \cdot \nabla Tdx=\int_{\Omega }v\beta_{\mathrm{diff}}\bar{\sigma }{\dot{\bar{\varepsilon }}}^{p}dx.$$

This derivation implicitly assumes that the material is isotropic with respect to the thermal conductivity λ. Using the Gaussian divergence theorem and defining the source term ζ as

(72) $$\begin{array}{cc} \zeta & =\zeta \left({\bar{\varepsilon }}^{p},{\dot{\bar{\varepsilon }}}^{p},T\right)=\beta_{\mathrm{diff}}\bar{\sigma }{\dot{\bar{\varepsilon }}}^{p}=\frac{\frac{\partial \psi *}{\partial {\dot{\bar{\varepsilon }}}^{p}}}{\bar{\sigma }}\bar{\sigma }{\dot{\bar{\varepsilon }}}^{p}=\frac{\partial \psi *}{\partial {\dot{\bar{\varepsilon }}}^{p}}{\dot{\bar{\varepsilon }}}^{p}\\ \; & =\left(A_{d}+B_{d}{\left({\bar{\varepsilon }}^{p}\right)}^{n}+\left(A+B{\left({\bar{\varepsilon }}^{p}\right)}^{n}\right)C\ln\frac{{\dot{\bar{\varepsilon }}}^{p}}{\varepsilon_{0}}\right)\left(1-\theta^{q}\right){\dot{\bar{\varepsilon }}}^{p}, \end{array}$$

as well as still using the definition of the nondimensional temperature θ from Equation (34), yields

(73) $$\int_{\Omega }v\rho_{0}c_{p}\frac{\partial T}{\partial t}dx+\int_{\Omega }\lambda \nabla v\cdot \nabla Tdx-\int_{\Gamma }v\lambda \nabla T\cdot nd\gamma =\int_{\Omega }v\zeta dx.$$

The entire Γ boundary is assumed to be an adiabatic Neumann boundary with ∇T·n=0. Thus, the corresponding term vanishes in Equation (73). A more detailed treatment of the boundary conditions will follow at the end of this section. A corresponding finite element discretization in index notation yields

(74) $$\begin{array}{cc} \sum_{i} & \sum_{j}{\hat{v}}_{i}\left[\int_{\Omega }\rho_{0}c_{p}N^{i}\left(x\right)\frac{\partial N^{j}\left(x\right){\hat{T}}_{j}}{\partial t}dx+\int_{\Omega }\lambda \nabla N^{i}\left(x\right)\cdot \nabla N^{j}\left(x\right)dx{\hat{T}}_{j}\right]\\ \; & =\sum_{i}{\hat{v}}_{i}\int_{\Omega }N^{i}\left(x\right)\zeta dx, \end{array}$$

with shape functions N, thus resulting in $T=N\cdot \hat{T}$. $\hat{T}$, which are the corresponding discrete values of the temperature field *T*. The same is true for $\hat{v}$. A rearrangement, and the fact that Equation (74) must hold for all arbitrary values of the weighting functions *v*, leads to

(75) $$\begin{array}{cc} \sum_{j}[ & \int_{\Omega }\rho_{0}c_{p}N^{i}\left(x\right)N^{j}\left(x\right)dx\frac{\partial {\hat{T}}_{j}}{\partial t}+\int_{\Omega }\lambda \nabla N^{i}\left(x\right)\cdot \nabla N^{j}\left(x\right)dx{\hat{T}}_{j}]\\ \int_{\Omega }N^{i}\left(x\right)\zeta dx=0\;\forall \;i. \end{array}$$

Applying a Gaussian quadrature, including a transformation from the physical space x∈Ω to the reference space $\xi \in \Omega^{\left(ref\right)}$, and summing over all elements results in

(76) $$\begin{array}{cc} \sum_{e}\sum_{k}[\sum_{j}( & w_{k}\rho_{0}c_{p}\left(\xi_{k}\right)N^{i}\left(\xi_{k}\right)N^{j}\left(\xi_{k}\right)\det J\left(\xi_{k}\right)\frac{\partial {\hat{T}}_{j}}{\partial t_{j}}\\ \; & +w_{k}\lambda \left(\xi_{k}\right)\left[J^{-T}\left(\xi_{k}\right)\nabla_{\xi }N^{i}\left(\xi_{k}\right)\right]\left[J^{-T}\left(\xi_{k}\right)\nabla_{\xi }N^{j}\left(\xi_{k}\right)\right]\det J\left(\xi_{k}\right){\hat{T}}_{j})]\\ \; & -w_{k}N^{i}\left(\xi_{k}\right)\zeta \left(\xi_{k}\right)\det J\left(\xi_{k}\right)=0\;\forall \;i. \end{array}$$

The indices *e* and *k* indicate that the functions and parameters are evaluated and taken, respectively, for the corresponding element or Gaussian integration point. Similarly, $w_{k}$ is the Gaussian weight for the integration point *k*. The functions $c_{p}\left(T\right),\lambda \left(T\right)$, and $\zeta ({\bar{\varepsilon }}^{p},{\dot{\bar{\varepsilon }}}^{p},T)$ are defined as follows

(77) $$\begin{array}{cc} c_{p}\left(\xi_{k}\right) & =c_{p}\left(N\left(\xi_{k}\right)\cdot {\hat{T}}_{old}\right), \end{array}$$

(78) $$\begin{array}{cc} \lambda (\xi_{k}) & =\lambda (N\left(\xi_{k}\right)\cdot {\hat{T}}_{old}), \end{array}$$

(79) $$\begin{array}{cc} \zeta (\xi_{k}) & =\zeta (N\left(\xi_{k}\right)\cdot {\hat{\bar{\varepsilon }}}^{p},N\left(\xi_{k}\right)\cdot {\hat{\dot{\bar{\varepsilon }}}}^{p},N\left(\xi_{k}\right)\cdot {\hat{T}}_{old}). \end{array}$$

The nomenclature ${\hat{\bar{\varepsilon }}}^{p}$ and ${\hat{\dot{\bar{\varepsilon }}}}^{p}$ were used to indicate the discrete nature of strains and strain rates, respectively. The measurement data from experiments eventually yielded a discrete field of displacements, strains, and strain rates at a set of points in the region of interest. This set of points served as the nodes in the FEM approach. The subdivision of this discrete field into quadrilateral elements, as used throughout this work, was performed with a basic algorithm that subsequently numbers and assigns the nodes in both spatial directions. It should be noted that the condition of the overall resulting system of equations can be improved in future research by using a more sophisticated approach to this discretization.

As a further side note, it may be advisable to replace the Jacobian determinant in Equation (76) by its absolute value $\left|\det J(\xi_{k})\right|$ for the purpose of the actual implementation, since this prevents the calculation from being affected if the transformation into parameter space unintentionally reverses the orientation of the axes. It should also be noted that, since quadrilateral elements and bilinear shape functions are used throughout this work, two Gaussian integration points per spatial direction were sufficient to ensure the accuracy of the integration.

The use of ${\hat{T}}_{old}$ in Equations (77)–(79) should only emphasize the above introduced approximation that the functions do not depend on the temperature ${\hat{T}}_{n+1}$ to be determined. Which temperatures are actually used depends on the time integration method used. The one used in this paper to solve an ordinary differential equation (ODE) of the form $\frac{\partial T}{\partial t}=f\left(T\left(t\right),t\right)$ was a classical one-step theta (OST) method, which resolves to [60] (pp. 312–315)

(80) $$T_{n+1}=T_{n}+\Theta \Delta tf\left(T_{n+1},t_{n+1}\right)+\left(1-\Theta \right)\Delta tf\left(T_{n},t_{n}\right),$$

where Θ is a numeric parameter to be specified later. Where Equation (76) is an ODE of the form $M\frac{\partial \hat{T}}{\partial t}=B\hat{T}+C$, the OST finally leads to a linear system of equations like

(81) $$\left(M_{n+1}-\Theta \Delta tB_{n+1}\right){\hat{T}}_{n+1}=M_{n}{\hat{T}}_{n}+\Theta \Delta tC_{n+1}+\left(1-\Theta \right)\Delta t\left(B_{n}{\hat{T}}_{n}+C_{n}\right),$$

where the coefficients are calculated as

(82) $$\begin{array}{cc} \left\{M_{n+1},B_{n+1},C_{n+1}\right\} & =f(x_{n+1},{\bar{\varepsilon }}_{n+1}^{p},{\dot{\bar{\varepsilon }}}_{n+1}^{p},{\hat{T}}_{n})\;\mathrm{and} \end{array}$$

(83) $$\begin{array}{cc} \left\{M_{n},B_{n},C_{n}\right\} & =f(x_{n},{\hat{\bar{\varepsilon }}}_{n}^{p},{\hat{\dot{\bar{\varepsilon }}}}_{n}^{p},{\hat{T}}_{n}). \end{array}$$

The use of ${\hat{T}}_{n}$ in Equation (82) was again due to the approximate assumption made to avoid the nonlinearity. For the choices Θ=0 and Θ=1 of the numerical parameter, the OST method was found to be essentially a forward (explicit) and a backward (implicit) Euler method, respectively. Choosing Θ=0.5 in the OST method resulted in the Crank–Nicolson method [61]. This method is particularly popular for solving diffusion equations, such as the heat equation of [62]. Its popularity is due to its stability and improved accuracy compared to the forward or backward Euler methods [63] (pp. 29–31, 237). Again, in considering the approximate nature of Equation (82), Θ=0.5 does not exactly yield a Crank–Nicolson approach. Nevertheless, it will remain the value of choice within this work.

This section concludes with some remarks on the physical boundary conditions and their theoretical incorporation. Since the deformation was given, no mechanical boundary conditions were imposed. For the temperature distribution, however, thermal boundary conditions (BCs) were found to be crucial to build a realistic model. For example, heat conduction within the specimen but outside the ROI can be approximated using a Robin boundary condition. Its introduction, which is also accompanied by some further considerations on its incorporation into the FEM approach, can be found in Appendix A.7. This extension of the mesh was again assumed to be adiabatic with respect to its other boundaries. Thus, the neglect of the boundary integral in Equation (73) is still valid.

## 7. Discussion and Conclusions

The energy-based variational model, which was introduced in Section 4 and evaluated in Section 5, is a promising approach through which to model the constitutive behavior in a thermodynamically consistent way. In particular, its modularity and its extension by arbitrary potentials to include other physical phenomena, such as damage, are attractive features. However, its fully implicit and symmetric formulation, which ensures unconditional stability and excellent convergence properties, as claimed by Stainier and Ortiz [27], are diminished by its adaptation to given displacement fields. These advantages are also offset by the significant computational disadvantages of the implementation in its current form, i.e., as a nonlinear optimization, when compared to the presented alternative approach. Furthermore, the apparent advantage of the EBVM in not using an explicit modeling of the TQC or $\beta_{\mathrm{diff}}$ factor is of limited significance as it is only replaced by the choice of the formulation of the plastic stored energy $W^{p}$ and the dual dissipation pseudo-potential ψ*, i.e., the respective splitting of the stress σ in Equation (35). However, the main disadvantage of the EBVM is clearly its apparent inconsistency and limited validity for the temperature-dependent modeling of the stored plastic energy $W^{p}\left(T\right)$, as discussed in Section 5.

The alternative approach, which was introduced in Section 6, is a fast, efficient, and comprehensible way through which to determine the temperature evolution for given displacement fields. However, the ALT approach itself is not fully consistent because it approximates the nonlinearity of heat Equation (70) by using temperatures from previous time steps for the source term in Equation (79). It also lacks a full theoretical derivation based on thermodynamic fundamentals and must therefore be considered an empirically motivated approach.

In conclusion, this work shows that the energy-based variational model seems to correctly and consistently describe material behavior only in the special case where the stored energy $W^{p}$ in the plastic is independent of temperature. An exhaustive theoretical investigation of this question is still pending. Furthermore, an efficient and tractable alternative approach for the determination of the temperature evolution for given displacement fields is proposed.

## 8. Outlook

Regardless of whether the EBVM or ALT approach is pursued, several issues need to be addressed to improve the accuracy of the calculated temperature evolution. First, the influence of macroscopic friction should be incorporated into the respective models for a more general analysis of different experiments or processes. One approach would be to include an accompanying mechanical simulation that provides, for example, any contact forces, shear, and slip velocity in the experiment from which the displacement field data are taken. From these values, a thermal boundary condition could be derived for a purely thermal ALT approach.

Further work could also consider elasticity. This area of research is less about the direct influence of the Joule–Thomson effect on the temperature evolution but more about the high values of hydrostatic pressure encountered. A separation of this hydrostatic pressure could allow for a distinction between elastic and plastic strains. This could lead to a more accurate evaluation of the stress state associated with the dissipative part of the irreversible deformation work, thus resulting in a temperature evolution.

Furthermore, the range of materials studied could be extended, and the EBVM and ALT approaches could also be applied to other constitutive models in a relatively straightforward manner.

Regarding the energy-based variational model, the obvious next step is the theoretical evaluation of the root cause of the problems arising for temperature-dependent plastic-stored energy terms $W^{p}\left(T\right)$. If it is not possible to eliminate the root cause, it might be recommendable to evaluate alternative incremental variational formulations, starting from the continuous formulation proposed by Yang et al. [24], e.g., the one published in Canadija and Mosler [64] (albeit they used internal energy instead of free energy). According to Stainier [46], this type of approach leads to a significantly higher complexity in the construction of analytical expressions. If the root cause can be eliminated, a next step might be a full derivation with respect to the temperature, as shown in Equation (42), and this would be accompanied by a linearization and a corresponding implementation with an FEM or Rayleigh–Ritz approach to reduce the computational effort. As a side note, a nonlinear FEM approach for the EBVM was developed and implemented during the preparation of this work. Its results were close to those obtained with the ALT approach for a stored plastic energy term that was independent of temperature. However, the computational efficiency was significantly inferior to the ALT approach due to the incorporated nonlinearity.

With a valid EBVM, another possible next step would be to extend it to other constitutive phenomena, e.g., to include a damage potential. This damage potential could be in the style of Lemaitre, who introduced a description of damage mechanics based on a scalar damage variable *D* ranging between D=0 for the undamaged state and D=1 for the rupture of the respective volume elements. A corresponding translation into potentials has been published by Lemaitre et al [45] (pp. 350, 396–409). In the present version of the EBVM and the ALT approach, the softening effect of material damage was implicitly and undifferentiatedly included by calibration with actual experimental results.

Furthermore, the EBVM in its present form for the use of displacement field data could be extended toward dynamics. A basis for such an adaptation is provided by Stainier [34].

Since the theoretical root cause analysis for the shortcomings of the EBVM is still missing, an efficient and comprehensible alternative approach is presented in Section 6. The most important next step is the establishment of a solid theoretical foundation since the ALT approach in its current form is, rather, an empirically motivated approach. However, this may be a by-product of the in-depth theoretical evaluation of the EBVM. Furthermore, for the sake of consistency, the source term in Equation (79) should be evaluated with a dependence on the correct temperature, i.e., it should not be approximated by using the temperature of a previous time step. In its current form, this would introduce a nonlinearity for the general case of the JC parameter q≠1. In order to avoid an unreasonable increase in the computational effort, a corresponding linearization is recommendable. Although a holistic calibration—i.e., including the thermal material behavior, which is based exclusively on stress–strain curves and may be a central advantage of the presented approach—its mathematical implications, especially with respect to the suspected local minima, should be subjected to a more profound theoretical analysis. Here, the experimental work on the time-resolved identification of the temperature-dependent plastic-stored energy terms $W^{p}\left(T\right)$ is especially promising for the purpose of further validating the approaches.

With regard to practical applications, the presented approaches take up the increasing capabilities of optical measurement systems for displacement and strain field identification. Using the presented methods enables one to directly derive parameters for coupled thermomechanical problems, e.g., in manufacturing processes like metal forming or machining.

## Figures and Tables

**Table 1:** Stainier–Ortiz material properties for the rate-independent 2023-T3 aluminum alloy from Stainier and Ortiz [27].

$\sigma_{0}\left(T_{0}\right)\;\left[\mathrm{MPa}\right]$	$\omega_{0}\;\left[K^{-1}\right]$	b[−]	n[−]	d[−]
255.0	0.0	−1.0	1.5	12.0
$\sigma_{1}\left(T_{0}\right)\;\left[\mathrm{MPa}\right]$	$\omega_{1}\;\left[K^{-1}\right]$	b′[−]	n′[−]	d′[−]
125.0	0.0007	4.0	1.0	−
${\hat{\sigma }}_{0}\left(T_{0}\right)\;\left[\mathrm{MPa}\right]$	${\hat{\omega }}_{0}\;\left[K^{-1}\right]$	${\hat{\sigma }}_{1}\left(T_{0}\right)\;\left[\mathrm{MPa}\right]$	${\hat{\omega }}_{1}\;\left[K^{-1}\right]$	m[−]
170.0	0.0	0.0	−	−
$\sigma_{\nu }\left(T_{0}\right)\;\left[\mathrm{MPa}\right]$	$\omega_{\nu }\;\left[K^{-1}\right]$	${\dot{\varepsilon }}_{0}\;\left[s^{-1}\right]$		
0.0	−	−		

**Figure A1:**
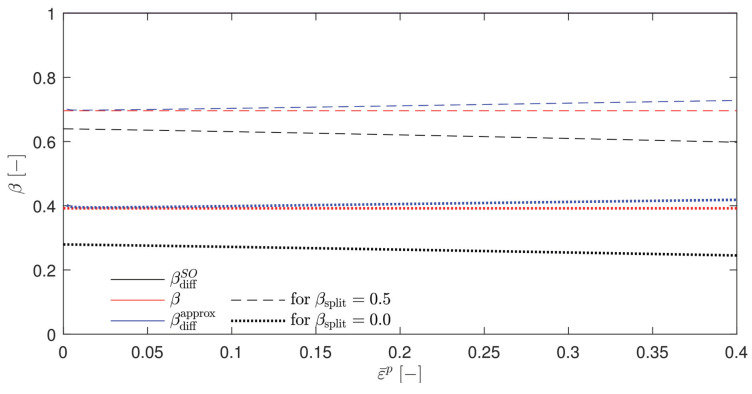
The β factors corresponding to the temperature evolution examined in Section 5.1. The color code describes the approach and the line code the respective value for β_split_.

**Figure A2:**
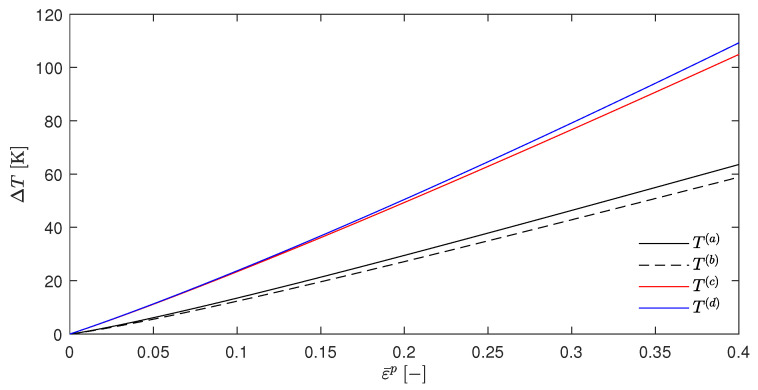
Temperature rise for $T^{\left(a\right)}$ (EBVM), $T^{\left(b\right)}$ ($\beta_{\mathrm{diff}}^{SO}$), $T^{\left(c\right)}$ (β), and $T^{\left(d\right)}$ ($\beta_{\mathrm{diff}}^{\mathrm{approx}}$) with $\beta_{\mathrm{split}}=0$ and q=0.9, which were examined with the approach and parameters that were introduced in Section 5.1.

**Figure A3:**
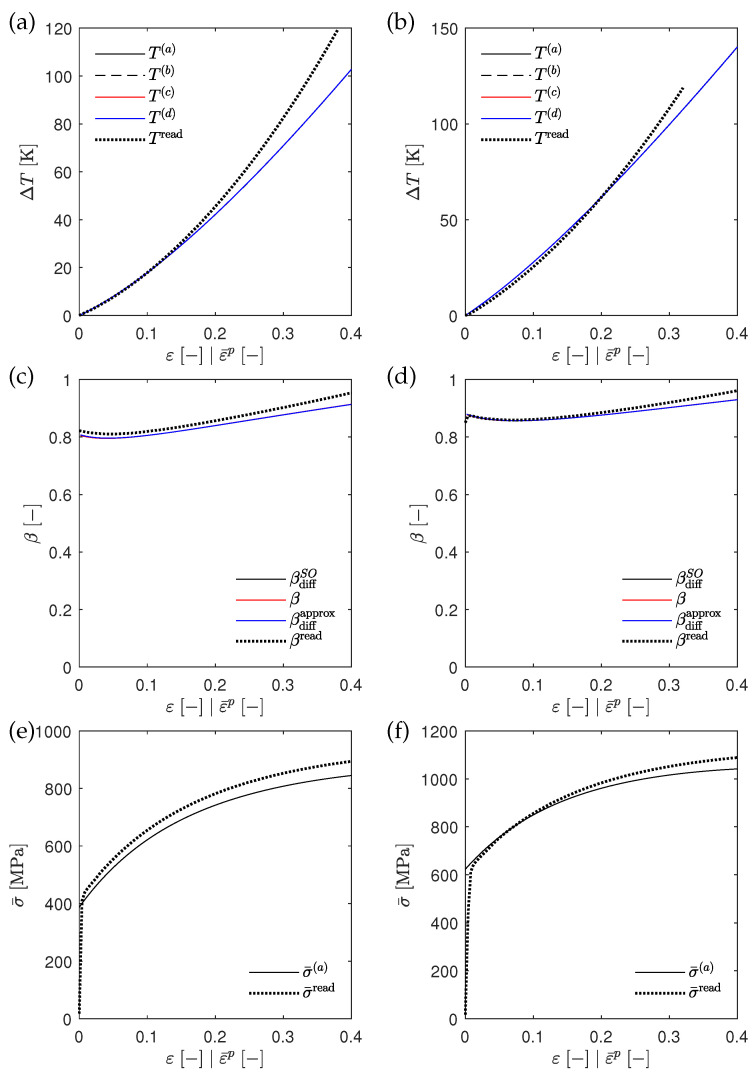
Comparison visualization of the calculated results against the results reported by Stainier and Ortiz [27] for (**a**,**b**) the temperature rise ΔT, (**c**,**d**) the β factors, and (**e**,**f**) the equivalent stress $\bar{\sigma }$ for the α-titanium in (**a**,**c**,**e**) a static (${\dot{\bar{\varepsilon }}}^{p}=1\times 10^{-3}\;s^{-1}$) and (**b**,**d**,**f**) a dynamic (${\dot{\bar{\varepsilon }}}^{p}=3000\;s^{-1}$) experiment.

## Data Availability

The data presented in this study are available on request from the corresponding author (accurately indicate status).

## Appendix A

### Appendix A.1. Derivation of the Resulting Adiabatic Heat

This section contains mathematical considerations on the derivation of the resulting adiabatic heat equation in the context of the EBVM for two different cases. Both are based on the assumption that the change in internal energy $\dot{U}$ is exclusively due to the current rate of irreversible deformation ${\dot{A}}^{p}=\bar{\sigma }{\dot{\bar{\varepsilon }}}^{p}$, i.e., there is neither heat transfer across boundaries nor additional source terms.

In the first case, it was assumed that there was no separate Helmholtz free energy, i.e., $W=W^{p}$. When utilizing the usual equation $\rho c_{p}=T\frac{\partial N}{\partial T}$ [65] (p. 141), the adiabatic heat equation can be derived as

σ¯ε¯˙p=U˙=W˙+TN˙+T˙NwithN=−∂W∂T=W˙+T∂N∂TT˙+T∂N∂ε¯pε¯˙p−T˙∂W∂Twithρcp=T∂N∂T=∂Wp∂TT˙+∂Wp∂ε¯pε¯˙p+ρcpT˙−T∂2Wp∂T∂ε¯pε¯˙p−∂Wp∂TT˙=σ¯Wpε¯˙p+ρcpT˙−T∂σ¯Wp∂Tε¯˙pρcpT˙=(T∂σ¯Wp∂T+σ¯ψ*)ε¯˙p.

The term multiplied by ${\dot{\bar{\varepsilon }}}^{p}$ is exactly the factor $\beta_{\mathrm{diff}}^{SO}$. In the second case, there was thermal Helmholtz free energy (as was the case in the EBVM), i.e., $W=W^{p}+W^{th}$. The adiabatic heat equation can then be written as

σ¯ε¯˙p=U˙=W˙+TN˙+T˙N=∂W∂TT˙+∂W∂ε¯pε¯˙p+T∂W∂T˙−∂W∂TT˙=∂Wp∂ε¯pε¯˙p+∂Wth∂ε¯pε¯˙p+T∂2W∂T2T˙+T∂2W∂T∂ε¯pε¯˙pwith∂2Wth∂T2=−ρcp1T=σ¯Wpε¯˙p+T∂2Wp∂T2T˙−ρcpT˙+T∂2Wp∂T∂ε¯pε¯˙p+T∂2Wth∂T∂ε¯pε¯˙pρcpT˙=(T∂σ¯Wp∂T+σ¯ψ*)ε¯˙p+T∂2Wp∂T2T˙.

Without further expansion of the term, it was stated that $T\frac{\partial^{2}W^{p}}{\partial T^{2}}\dot{T}=0\;\forall \;q=1$. Thus, this additional term could be the source of the observed discrepancies for the general case of q≠1.

### Appendix A.2. Derivation of Equivalent Strain and Stress for Uni-Axial Loading

For the strain rates, the explicit relationship between the true form of the variables and the respective von Mises equivalent form with respect to plastic strains and strain rates is given (which are related to [66] (p. 19)):

(A1) $$\begin{array}{c} {\dot{\bar{\varepsilon }}}^{p}=\sqrt{\frac{2}{3}{\dot{\varepsilon }}_{ij}^{p}{\dot{\varepsilon }}_{ij}^{p}}=\sqrt{\frac{2}{3}\left[{\left({\dot{\varepsilon }}_{11}^{p}\right)}^{2}+{\left({\dot{\varepsilon }}_{22}^{p}\right)}^{2}+{\left({\dot{\varepsilon }}_{33}^{p}\right)}^{2}\right]+\frac{4}{3}\left[{\left({\dot{\varepsilon }}_{23}^{p}\right)}^{2}+{\left({\dot{\varepsilon }}_{31}^{p}\right)}^{2}+{\left({\dot{\varepsilon }}_{12}^{p}\right)}^{2}\right]}. \end{array}$$

In the case of uniaxial loading and an associated flow rule, the strain rate components can be expressed as ${\dot{\varepsilon }}_{11}^{p}=\dot{\varepsilon^{p}}$, ${\dot{\varepsilon }}_{22}^{p}={\dot{\varepsilon }}_{33}^{p}=-\nu {\dot{\varepsilon }}_{11}^{p}$ and ${\dot{\varepsilon }}_{23}^{p}={\dot{\varepsilon }}_{31}^{p}={\dot{\varepsilon }}_{12}^{p}=0$. Assuming further that the plastic flow is isochoric, i.e., has a Poisson ratio ν=0.5, we obtain

(A2) $$\begin{array}{c} {\dot{\bar{\varepsilon }}}^{p}=\sqrt{\frac{2}{3}\left[{\left({\dot{\varepsilon }}^{p}\right)}^{2}+{\left(-\frac{1}{2}{\dot{\varepsilon }}^{p}\right)}^{2}+{\left(-\frac{1}{2}{\dot{\varepsilon }}^{p}\right)}^{2}\right]}=\left|{\dot{\varepsilon }}^{p}\right|. \end{array}$$

Starting with this equation and using two basic identities—$\varepsilon^{p}=\int {\dot{\varepsilon }}^{p}dt$ and ${\bar{\varepsilon }}^{p}=\int {\dot{\bar{\varepsilon }}}^{p}dt$—as well as the fact that there is no change in sign of the plastic strain rate ${\dot{\varepsilon }}^{p}$ under uniaxial loading, it can be stated that ${\bar{\varepsilon }}^{p}=\varepsilon^{p}$.

Considering the work conjugation, i.e., $\sigma {\dot{\varepsilon }}^{p}=\bar{\sigma }{\dot{\bar{\varepsilon }}}^{p}$ [66] (p. 27), Equation (A2) also implies the identity $\bar{\sigma }=\sigma$. Thus, the equivalent stress–strain curves were found to be identical to the original stress–strain curves for the case of uniaxial loading. For the sake of brevity, it should be emphasized that the above considerations are by no means an exhaustive mathematical proof; rather, they should be seen as considerations from an engineering point of view.

### Appendix A.3. Examined Influences on the Issues of the Energy-Based Variational Model

This section lists some minor adjustments to the underlying calculations used to generate the results shown throughout the Section 5, which have no significant impact on the overall shape, i.e., they cause a relative change in the final maximum temperature rise $\max(\Delta T\left(t_{\mathrm{end}}\right))$ of less than 2%. These settings are the choice of the following:

- The algorithmic parameter α∈[0,1] in Equation (32);
- The optimization solver in The Mathworks, Inc. [55], i.e., fminunc or fminsearch;
- The respective starting points for these solvers;
- The time integration scheme, i.e., what was introduced in Section 5, or a forward Euler method;
- The specific form of the integral calculation $\int_{t_{n}}^{t_{n+1}}\psi *\prime dt\prime$. This can be conducted either by using Equation (31) or as $\int_{t_{n}}^{t_{n+1}}\psi *\prime dt\prime =\Delta t\psi *\left(\frac{T_{n+1}}{T_{n}}{\dot{\bar{\varepsilon }}}_{n+1}^{p},{\bar{\varepsilon }}_{n}^{p},T_{n}\right)$ [42] (p. 63).

Furthermore, the general form of the discrepancies between $T^{\left(a\right)}$ and $T^{\left(d\right)}$ was found to be independent of the set of JC constitutive parameters as long as the EBVM converges at all.

### Appendix A.4. β Plots for the Review of the Energy-Based Variational Model with the Johnson–Cook Constitutive Model

Figure A1 shows the β factors corresponding to the temperature evolution from $T^{\left(a\right)}$ to $T^{\left(d\right)}$, as studied in Section 5.1. The fact that β is constant throughout the deformation is due to the equal splitting of $\frac{A_{d}}{A}=\frac{B_{d}}{B}$. With this splitting, Equation (45) is reduced to

(A3) β=Ad+Bd(ε¯p)n+(A+B(ε¯p)n)Clnε¯˙pε0(A+B(ε¯p)n)(1+Clnε¯˙pε0)=βsplit(A+B(ε¯p)n+(A+B(ε¯p)n)Clnε¯˙pε0(A+B(ε¯p)n)(1+Clnε¯˙pε0)=βsplit+Clnε¯˙pε01+Clnε¯˙pε0

### Appendix A.5. Temperature Evolution for the Review of the Energy-Based Variational Model with the Johnson–Cook Constitutive Model with Parameter q ≠ 1

Figure A2 shows the temperature increases for the temperatures $T^{\left(a\right)}$ to $T^{\left(d\right)}$, as calculated in Section 5.1 with $\beta_{\mathrm{split}}=0$ and q=0.9. It can be clearly seen that the temperatures $T^{\left(a\right)}$ and $T^{\left(b\right)}$ were no longer congruent in the case q≠1.

### Appendix A.6. Stainier–Ortiz Model for an α-Titanium Alloy

Ref. Stainier and Ortiz [27] also reported results for the study of a α titanium alloy with their version of the energy-based variational model. The data were for experiments at an initial temperature of $T_{0}=293\;K$, which was achieved using the general material parameters for specific heat capacity $c_{p}=518.0\;{\mathrm{Jkg}}^{-1}K^{-1}$ and density $\rho_{0}=4500.0\;\mathrm{kg}\;m^{-3}$. The constitutive parameters are listed in the Table A1.

**Table A1:** Material properties for the rate-dependent α-titanium alloy from Stainier and Ortiz [27].

$\sigma_{0}\left(T_{0}\right)\;\left[\mathrm{MPa}\right]$	$\omega_{0}\;\left[K^{-1}\right]$	b[−]	n[−]	d[−]
75.0	0.0	−4.0	1.0	10.0
$\sigma_{1}\left(T_{0}\right)\;\left[\mathrm{MPa}\right]$	$\omega_{1}\;\left[K^{-1}\right]$	b′[−]	n′[−]	d′[−]
300.0	0.0008	5.0	1.5	7.0
${\hat{\sigma }}_{0}\left(T_{0}\right)\;\left[\mathrm{MPa}\right]$	${\hat{\omega }}_{0}\;\left[K^{-1}\right]$	${\hat{\sigma }}_{1}\left(T_{0}\right)\;\left[\mathrm{MPa}\right]$	${\hat{\omega }}_{1}\;\left[K^{-1}\right]$	m[−]
120.0	0.0	200.0	0.0	5.0
$\sigma_{\nu }\left(T_{0}\right)\;\left[\mathrm{MPa}\right]$	$\omega_{\nu }\;\left[K^{-1}\right]$	${\dot{\varepsilon }}_{0}\;\left[s^{-1}\right]$		
50.0	0.0006	1.0		

The comparisons of the temperature rises ΔT, the β factors, and the equivalent stresses $\bar{\sigma }$ are shown in Figure A3a,c,e for a static experiment, as well as in Figure A3b,d,f for a dynamic experiment.

The aforementioned figures were designed in a similar way to Figure 4, Figure 5, Figure 6, Figure 7 and Figure 8. For a detailed description, the interested reader is referred to Section 5.2. It should also be noted that Figure A3 serves only to demonstrate the similarity of the EBVM version, as was adapted and used in the present work, to the calculations of Stainier and Ortiz [27]. Changing the parameters again in a way that the stored plastic energy term $W^{p}$ becomes temperature-dependent was deliberately avoided. However, it was noted that, in this case, there was again a significant discrepancy between the evolution of the temperatures $T^{\left(a\right)}$ and $T^{\left(d\right)}$.

### Appendix A.7. On Possible Boundary Conditions

Throughout the present work, the conductive heat transfer from the ROI of the recorded displacement field data into the rest of the sample was considered by extending the grid. In the case of the spatial resolution of the measured input data becoming too high or if a computationally more expensive approach is used, it is recommended to replace this extension with a more efficient but less accurate explicit modeling of the respective boundary condition.
